# An Entropy-Regularised AI Framework for Multi-Asset Volatility Spillover Forecasting and CVaR-Constrained Portfolio Allocation in Financial Markets

**DOI:** 10.3390/e28070756

**Published:** 2026-07-01

**Authors:** Jiawei Yu, Lu Wang, Xinyan Sun

**Affiliations:** 1 School of Finance, Anhui University of Finance and Economics, Bengbu 233030, China; 20234098@aufe.edu.cn; 2 Business School, Massey University, Palmerston North 4442, New Zealand; 23010652@massey.ac.nz; 3 Faculty of Computer and Mathematical Sciences, The Hong Kong Polytechnic University, Hong Kong 999077, China

**Keywords:** transfer entropy, variational information bottleneck, graph attention network, volatility spillover, deep learning, uncertainty quantification, CVaR, tail risk

## Abstract

Forecasting multi-asset volatility spillovers and turning the forecasts into risk-aware portfolios requires methods that uncover directional information flow between assets, compress the state into a minimal sufficient representation, deliver calibrated uncertainty, and respect explicit tail-risk limits. We propose TDV (Transfer-entropy, Dynamic-graph-attention, Variational-information-bottleneck), an information-theoretic artificial intelligence framework that couples a time-varying transfer entropy network with a graph attention encoder regularised by a variational information bottleneck, and demonstrates the practical value of the calibrated predictive distribution through a downstream entropy-regulated, CVaR-constrained portfolio application. We establish three theoretical results: $L^{2}$ consistency of the *k*-nearest-neighbour transfer entropy estimator on α-mixing returns with rate $O_{P}\left(n^{-2/(2+d)}\right)$, a PAC–Bayes generalisation bound of order $O(\sqrt{(I(X;Z)+\log(1/\delta ))/n})$ for the bottleneck-encoded forecaster, and asymptotic CVaR feasibility of the plug-in allocation. In simulations across sparse Granger networks, contagion DCC–GARCH ensembles, and regime-switching factor models, the framework cuts spillover forecasting errors by 24 to 42 percent against LSTM, vanilla GAT, and Transformer baselines, and it recovers 1.6 additional nats of mutual information with the realised connectedness matrix. On a 32-asset global panel covering 2014 to 2025, the model delivers an out-of-sample $R^{2}$ of 0.331, an annualised Sharpe ratio of 1.46 against 0.83 for an equally weighted benchmark, a maximum drawdown of 7.8 percent, and 95 percent CVaR reductions of 28 to 36 percent across sub-periods relative to a shrinkage minimum-variance baseline.

## 1. Introduction

Forecasting volatility and managing tail risk across a multi-asset universe is one of the central problems in quantitative finance. Classical univariate models such as GARCH [1] and HAR [2] deliver parsimonious point forecasts of conditional variance, while multivariate extensions such as DCC–GARCH [3] and the Diebold–Yilmaz spillover index [4] provide useful descriptive measures of cross-asset connectedness. These models, however, rely on linear, contemporaneous covariance structures, which cannot capture the directional, lagged, and nonlinear information flow that characterises modern financial markets and that empirical studies of crisis transmission have repeatedly documented [5].

From an information-theoretic viewpoint, the natural object that summarises directional dependence between two stochastic processes is the transfer entropy of Schreiber [6], which generalises Granger causality to nonlinear and non-Gaussian settings. Empirical applications to financial time series have shown that transfer entropy detects regime changes and contagion episodes that linear measures miss [7]. Estimating transfer entropy from finite samples is nontrivial; the nearest-neighbour estimator of Kraskov, Stögbauer, and Grassberger [8] provides a bias-corrected plug-in estimator with favourable finite-sample properties, but to the best of our knowledge it has not been combined with modern graph deep learning in a unified probabilistic framework that delivers uncertainty quantification and risk-aware portfolio rules.

In parallel, graph neural networks have emerged as a powerful tool for relational learning [9,10,11], with attention-based variants such as the Graphormer [12] and the graph transformer [13] matching or surpassing convolutional counterparts on a range of benchmarks. In finance, recent applications include stock prediction with temporal relational graphs [14,15] and relational learning for credit risk [16]. These efforts typically build the graph from precomputed correlations or sector membership, losing the directional information that transfer entropy would supply.

A second methodological pillar is the variational information bottleneck (VIB) of Alemi et al. [17], which extends the information bottleneck principle to deep networks by minimising the mutual information I(X;Z) between input *X* and latent *Z* subject to a constraint on the predictive information I(Z;Y), and which provides a tractable variational upper bound that has been linked to generalisation in deep networks [18,19]. Despite the active literature on information-theoretic representation learning [20], the combination of a VIB-regularised graph encoder with transfer entropy edges has not, to our knowledge, been studied in the context of multi-asset volatility forecasting and portfolio construction.

A third pillar is risk-aware portfolio construction. Conditional Value at Risk (CVaR) provides a coherent risk measure that admits a convex reformulation [21], and entropy-based portfolio construction [22] has shown that information-theoretic objectives can improve diversification beyond mean–variance. We unify these ideas: the predictive entropy from a calibrated forecaster acts as a position scaling signal, while the Kullback–Leibler divergence between the bottleneck posterior and a structured prior measures model risk and enters a CVaR-constrained second-order cone programme.

Three desiderata follow from this discussion. The model should build the inter-asset graph from directional, time-varying information measures rather than from sample correlations; the encoder should compress the input to a minimal sufficient representation, so that overfitting is controlled and the predictive distribution is well calibrated; and the resulting predictive distribution should be operationalised into a portfolio rule that respects explicit tail-risk constraints. The proposed TDV framework satisfies all three.

The primary contribution of this paper is the TDV forecasting model, which captures directional, nonlinear volatility spillovers with calibrated uncertainty through the transfer entropy graph, the graph attention encoder, and the variational information bottleneck (Section 2.2, Section 2.3 and Section 2.4). To demonstrate the practical value of the calibrated predictive distribution, we present a downstream portfolio application in which the uncertainty estimates are consumed by an entropy-regulated, CVaR-constrained allocation rule (Section 4). The CVaR-constrained programme itself employs the standard convex formulation of Rockafellar and Uryasev [21]; the contribution of Section 4 lies not in the optimisation technique but in the information-theoretic inputs—the predictive entropy penalty and the KL-based model risk measure—that the TDV forecaster supplies. The theoretical results (Theorems 1–3) cover both the forecasting model and its downstream application, confirming that calibrated uncertainty propagates correctly into tail-risk control.

Information-theoretic financial econometrics is by now a mature field [7,22], yet contemporary deep learning systems rarely treat differential entropy and KL divergence as first-class operational signals. The present paper closes that gap by coupling a graph attention forecaster with a VIB regulariser and by deriving theoretical guarantees that justify the use of the predictive entropy in the downstream allocation step.

Turning to deep learning for finance, the survey of [23] catalogued several hundred neural network applications to forecasting and trading; empirical studies by Gu, Kelly, and Xiu [24] and Fischer and Krauss [25] demonstrated that machine learning materially outperforms classical models for the cross section of returns and that deep hedging [26,27] extends the methodology to derivative risk management. These approaches enlarge the function class but typically output point estimates without principled uncertainty quantification. Bayesian neural networks [28], Monte Carlo dropout [29], and deep ensembles [30] approximate posteriors but typically lack coverage guarantees. The VIB layer adopted here provides closed-form predictive entropies under the Gaussian encoder, sidestepping the sampling cost while retaining a principled information-theoretic interpretation of regularisation.

Table 1 positions the proposed framework against representative prior studies along seven axes, including the theoretical guarantees that distinguish our contribution.

Table 1 shows that no single existing approach addresses all seven dimensions jointly. The contributions of this paper close that gap. The scope of the claimed contribution is deliberately circumscribed: transfer entropy estimation, graph attention, the variational information bottleneck, and CVaR optimisation are individually established methodologies, and the portfolio programme follows the standard convex formulation of Rockafellar and Uryasev [21]. The contribution lies in the integrated architecture, in the structural by-products that the integration uniquely enables (the entropy decomposition, the KL-based model risk measure, and the entropy-penalised allocation rule), and in the three theoretical results (Theorems 1–3) that certify the end-to-end pipeline. The three structural interactions that the integration creates are detailed below.

The contributions form a connected progression. The constituent components, transfer entropy estimation, graph attention networks, the variational information bottleneck, and CVaR optimisation, are individually established.

The novelty resides not in the individual components but in three structural interactions that the integrated architecture creates and that no proper subset of the ingredients can reproduce. The novelty manifests in three structural interactions. At the graph level, the injection of transfer entropy weights into the attention logits (Equation (9), via the learnable mixing coefficient β) endows the graph attention mechanism with a directional, nonlinear information-theoretic prior on the message passing geometry; this prior is absent from standard graph attention networks that learn attention from node features alone, and it cannot be recovered by post hoc thresholding of a learned attention matrix, because the transfer entropy signal shapes the gradient landscape during training rather than merely filtering the output. At the representation level, the variational information bottleneck serves a dual role that is unique to this architecture: it simultaneously regularises the encoder through the PAC–Bayes bound (Theorem 2), whose complexity term is the very KL penalty minimised during training, and it produces a closed-form Gaussian predictive distribution whose differential entropy decomposes into aleatoric and epistemic components (Proposition 2); this dual role is specific to the Gaussian bottleneck placed after the graph attention layers and does not arise in generic Bayesian neural networks, Monte Carlo dropout, or deep ensembles, all of which require sampling-based entropy estimates without closed-form decompositions. At the allocation level, the predictive entropy and the inter-specification KL divergence furnish information-theoretic inputs to the CVaR-constrained allocation that conventional plug-in variance estimates cannot supply: the entropy penalty (Equation (23)) scales each position by a measure of the model’s own calibrated uncertainty rather than by a point-estimate risk premium, and the CVaR feasibility guarantee (Theorem 3) ensures that the encoder’s calibration propagates to the tail-risk constraint, closing a theoretical loop between representation learning and portfolio feasibility that remains open when the two stages are treated as independent modules. Specifically, the proposed TDV framework:

1. Builds a directed, time-varying graph whose edge weights are bias-corrected *k*-nearest-neighbour estimates of pairwise transfer entropy on a rolling window, supplying directional and nonlinear information that Pearson and Spearman correlation networks miss (Section 2.2).
2. Learns node embeddings through a multi-head graph attention encoder whose attention scores are augmented by the transfer entropy weights, so that the information flow estimated in the data shapes the message passing geometry (Section 2.3).
3. Compresses the resulting representation through a variational information bottleneck layer that minimises the mutual information I(X;Z) while maximising the predictive mutual information I(Z;Y), with a closed-form Gaussian posterior that yields tractable predictive entropy and KL divergence (Section 2.4).
4. Establishes three theoretical results: $L^{2}$ consistency and an $O_{P}\left(n^{-2/(2+d)}\right)$ rate for the *k*-nearest-neighbour transfer entropy estimator under α-mixing (Theorem 1), a PAC–Bayes generalisation bound for the bottleneck-encoded graph attention forecaster (Theorem 2), and asymptotic feasibility of the CVaR-constrained allocation under the calibrated predictive distribution (Theorem 3). Each result addresses a gap in the existing literature that is not closed by the constituent techniques alone. Theorem 1 extends the *K*SG consistency proof from i.i.d. samples to α-mixing processes via Berbee’s coupling construction, yielding a convergence rate tailored to the serial dependence structure of financial returns. Theorem 2 derives a PAC–Bayes bound whose complexity term is the sample-averaged KL divergence of a graph-structured VIB encoder, linking the information bottleneck penalty directly to the generalisation gap in a non-i.i.d. setting. Theorem 3 completes the chain by proving that the convergence of the encoder’s predictive moments propagates through the CVaR functional to guarantee asymptotic constraint satisfaction, a result that requires the joint analysis of the encoder, the shrinkage estimator, and the portfolio optimiser and does not follow from any one component in isolation.
5. Demonstrates the practical utility of the calibrated predictions through a downstream portfolio application, in which the predictive differential entropy modulates each position via an uncertainty-aware exponential penalty, the KL divergence between the bottleneck posterior and a structured prior measures model risk, and a standard CVaR constraint is enforced as a second-order cone programme (Section 4).

The integrative point is that combining transfer entropy edges, graph attention, variational information bottleneck, and CVaR optimisation within one semiparametric model yields structural by-products, namely an entropy decomposition of forecast uncertainty, KL bounds on cross-specification divergence, and an entropy-penalised allocation rule that are simply not available when the ingredients are deployed separately. Only the full pipeline delivers all three: interpretable directional attention, closed-form entropy with an aleatoric–epistemic split, and a provable CVaR feasibility certificate, as established by the three structural interactions described above. The ablation study supplies direct empirical support for this claim: removing transfer entropy edges in favour of Pearson correlation raises MSFE by 33 percent, removing the VIB layer (γ=0) widens the gap between empirical and nominal coverage to 11 percentage points, and replacing the entropy modulation with a flat momentum signal reduces the Sharpe ratio by 0.23. Each degradation is attributable to exactly one missing link in the pipeline and cannot be compensated by the remaining components, confirming that the three structural interactions are individually necessary.

The experimental design tests the integrative claim. We report simulation studies under three canonical data generating processes (sparse Granger networks, contagion DCC–GARCH ensembles, and regime-switching factor models), adversarial misspecification studies, finite-sample calibration diagnostics, sub-period robustness on a global multi-asset panel, baseline hyperparameter tuning protocols, and a transaction cost sensitivity analysis. The proposed framework achieved the lowest mean squared forecasting error in every scenario, attained 94.2 percent empirical coverage of nominal 95 percent prediction intervals, and delivered a 1.46 annualised Sharpe ratio on the real data panel, with CVaR reductions of 28 to 36 percent over a minimum-variance benchmark and 22 to 28 percent over a vanilla graph attention baseline.

The remainder of the paper proceeds as follows. Section 2 constructs the TDV framework, covering the transfer entropy graph construction, the graph attention encoder, the variational information bottleneck, the joint training objective, and the supporting theoretical results. Section 3 develops the information-theoretic analysis, including the predictive entropy decomposition and its link to spillover and tail risk. Section 4 presents a downstream portfolio application based on entropy-regulated, CVaR-constrained allocation that demonstrates the practical value of the calibrated predictions. Section 5 presents the simulation and real data experiments. Section 6 discusses implications and limitations, and Section 7 concludes.

## 2. Transfer Entropy Dynamic Graph Attention Framework with Variational Information Bottleneck

### 2.1. Setup and Notation

Let (Ω,F,P) be a probability space carrying a complete, right-continuous filtration ${\left(F_{t}\right)}_{t\geq 0}$. Consider *N* financial assets with log price processes ${\left(P_{t,i}\right)}_{t\geq 0}$, i=1,…,N. Define daily log returns $r_{t,i}=P_{t,i}-P_{t-1,i}$, intraday realised variance ${\mathrm{RV}}_{t,i}$ computed from five-minute returns by the standard estimator, and daily realised volatility ${\mathrm{RVol}}_{t,i}=\sqrt{{\mathrm{RV}}_{t,i}}$.

The forecasting target is the *h*-step ahead realised volatility vector

(1) $$y_{t+h}={\left({\mathrm{RVol}}_{t+h,1},\;\ldots ,\;{\mathrm{RVol}}_{t+h,N}\right)}^{⊤}.$$

We write $y_{t+h,i}={\mathrm{RVol}}_{t+h,i}$ for the *i*-th component of $y_{t+h}$. The predictor at time *t* is a tuple $X_{t}=\left(X_{t},A_{t}\right)$, with $X_{t}\in R^{N\times d_{x}}$ a matrix of node features (lagged returns, realised variance components at multiple horizons, range-based volatility estimators, technical indicators, and macro factors), and $A_{t}\in R^{N\times N}$ a directed, weighted adjacency matrix encoding transfer entropy between assets over a rolling window. We let $D_{n}={\left\{\left(X_{t},y_{t+h}\right)\right\}}_{t=1}^{n}$ be the training set. To prevent ambiguity, we fix the following notational conventions for the remainder of the paper. Greek letters ϕ and θ always denote learnable parameters of the encoder and decoder, respectively; the standard normal probability density function is written φ(·) and the standard normal cumulative distribution function is written Φ(·). Starred quantities (e.g., ${\hat{\mu }}_{i}^{*}$, ${\hat{v}}_{i}^{*}$) refer to predictions evaluated at a test time point $t^{*}$. Bold uppercase letters denote matrices or vectors of vectors (e.g., $H_{t}^{\left(\ell \right)}\in R^{N\times d_{h}}$), while bold lowercase letters denote individual vectors (e.g., $h_{t,i}^{\left(\ell \right)}\in R^{d_{h}}$).

Because the paper spans information theory, graph learning, GARCH econometrics, and portfolio optimisation, several standard symbols inevitably serve more than one conventional role; Appendix A provides a complete list of symbols and their context-specific meanings.

### 2.2. Transfer Entropy Graph Construction

**Definition** **1** (Schreiber transfer entropy). *For any two assets i and j whose return processes ${\left\{r_{t,i}\right\}}_{t\in Z}$ and ${\left\{r_{t,j}\right\}}_{t\in Z}$ are strictly stationary (as defined in Section 2.1) the transfer entropy from j to i at lags (k,l) is*

(2) $$T_{j\to i}^{\left(k,l\right)}=\sum p\left(r_{t+1,i},r_{t}^{\left(k,i\right)},r_{t}^{\left(l,j\right)}\right)\log\frac{p\left(r_{t+1,i}|r_{t}^{\left(k,i\right)},r_{t}^{\left(l,j\right)}\right)}{p\left(r_{t+1,i}|r_{t}^{\left(k,i\right)}\right)},$$

*where $r_{t}^{\left(k,i\right)}=\left(r_{t,i},r_{t-1,i},\ldots ,r_{t-k+1,i}\right)$ and $r_{t}^{\left(l,j\right)}=\left(r_{t,j},r_{t-1,j},\ldots ,r_{t-l+1,j}\right)$. The sum is replaced by a Lebesgue integral for continuous distributions.*

Transfer entropy [6] is asymmetric, $T_{j\to i}\neq T_{i\to j}$, non-negative and reduces to standard Granger causality under Gaussianity. Unlike the Pearson correlation, it captures directional, nonlinear dependence; unlike Granger causality, it remains well defined for non-Gaussian and heavy-tailed return distributions.

#### 2.2.1. Bias-Corrected Nearest-Neighbour Estimator

Direct histogram estimation of Equation (2) suffers from severe bias and exponential variance in the embedding dimension. We adopt the *k*-nearest-neighbour estimator of Kraskov, Stögbauer, and Grassberger [8]. For a sample of size *n*, lags (k,l), and neighbour count *K*, the estimator is

(3) $${\hat{T}}_{j\to i}^{\left(k,l\right)}=\psi \left(K\right)-\psi \left({\bar{n}}_{1}\right)+\psi \left({\bar{n}}_{2}\right)-\psi \left({\bar{n}}_{3}\right),$$

where ψ is the digamma function. Denote by ϵ(s) the Chebyshev distance from sample point *s* to its *K*-th-nearest-neighbour in the joint space $u_{s}=\left(r_{t+1,i},\;r_{t}^{\left(k,i\right)},\;r_{t}^{\left(l,j\right)}\right)$. Let $n_{1}\left(s\right)$ be the number of points whose distance in the marginal subspace $r_{t}^{\left(k,i\right)}$ is strictly less than ϵ(s), let $n_{2}\left(s\right)$ be the analogous count in the subspace $\left(r_{t+1,i},\;r_{t}^{\left(k,i\right)}\right)$, and let $n_{3}\left(s\right)$ be the count in the subspace $\left(r_{t}^{\left(k,i\right)},\;r_{t}^{\left(l,j\right)}\right)$. Then ${\bar{n}}_{\ell }=\frac{1}{n}\sum_{s=1}^{n}\psi \left(n_{\ell }\left(s\right)+1\right)$ for ℓ=1,2,3. The bias correction subtracts a small sample mean estimate using Fourier surrogate data [7]: we generate S=100 Fourier surrogates of $\left\{r_{t,j}\right\}$ that preserve the marginal distribution and power spectrum but destroy phase information. We compute the surrogate transfer entropy mean

(4) $${\hat{T}}_{j\to i}^{\mathrm{surr}}=\frac{1}{S}\sum_{s=1}^{S}{\hat{T}}_{j\to i}^{\left(k,l\right)}\left(\left\{r_{t,i}\right\},\;\left\{r_{t,j}^{\left(s\right)}\right\}\right),$$

where each $\left\{r_{t,j}^{\left(s\right)}\right\}$ is a Fourier phase-randomised surrogate of $\left\{r_{t,j}\right\}$ that preserves the marginal distribution and power spectrum but destroys the phase coupling with $\left\{r_{t,i}\right\}$, and where ${\hat{T}}_{j\to i}^{\left(k,l\right)}\left(\cdot ,\cdot \right)$ denotes the KSG estimator of Equation (3) applied to the surrogate pair: the two arguments $\left\{r_{t,i}\right\}$ and $\left\{r_{t,j}^{\left(s\right)}\right\}$ are the full univariate return series for assets *i* and *j* (the latter being the *s*-th surrogate), from which the estimator internally constructs the lag-embedding vectors $r_{t}^{\left(k,i\right)}$ and $r_{t}^{\left(l,j\right)}$ as defined in Equation (2). We then report the effective transfer entropy

(5) $${\hat{T}}_{j\to i}^{\mathrm{eff}}=\max\left({\hat{T}}_{j\to i}^{\left(k,l\right)}-{\hat{T}}_{j\to i}^{\mathrm{surr}},\;0\right).$$

The KSG estimator (Equation (3)) and the Fourier surrogate correction (Equation (4)) address two distinct sources of bias. The KSG estimator corrects for finite-sample bias in the *k*-nearest-neighbour density estimation step, replacing histogram bin counts with adaptive neighbour counts that are approximately unbiased for the entropy functional. The Fourier surrogate correction addresses a separate problem: even with an unbiased density estimator, serial autocorrelation in financial returns induces spurious transfer entropy because lagged self-similarity in $\left\{r_{t,j}\right\}$ can mimic genuine information flow from *j* to *i*. The phase-randomised surrogates preserve the marginal distribution and the autocorrelation structure (power spectrum) of $\left\{r_{t,j}\right\}$ while destroying its temporal phase coupling with $\left\{r_{t,i}\right\}$, so that the surrogate mean ${\hat{T}}_{j\to i}^{\mathrm{surr}}$ estimates the spurious floor attributable to autocorrelation alone. Subtracting this floor and clipping at zero yields the effective transfer entropy (Equation (5)), which isolates genuine directional information flow. Both corrections are therefore necessary: omitting the KSG correction inflates variance, while omitting the surrogate correction inflates bias.

**Assumption** **1.**

(A1) *The joint return process ${\left\{\left(r_{t,1},\ldots ,r_{t,N}\right)\right\}}_{t\in Z}$ is strictly stationary (i.e., for every finite collection of indices $t_{1},\ldots ,t_{m}$ and every integer shift τ the joint distribution of $\left(r_{t_{1}},\ldots ,r_{t_{m}}\right)$ equals that of $\left(r_{t_{1}+\tau },\ldots ,r_{t_{m}+\tau }\right)$) and α-mixing, where the strong mixing coefficient is defined by $\alpha \left(k\right)=\sup_{A\in F_{-\infty }^{0},\;B\in F_{k}^{\infty }}\left|P\left(A\cap B\right)-P\left(A\right)P\left(B\right)\right|$ with $F_{a}^{b}=\sigma \left(r_{t}:a\leq t\leq b\right)$, with mixing coefficients verifying $\sum_{k=1}^{\infty }\alpha {\left(k\right)}^{\delta /(2+\delta )}<\infty$ for some δ>0.*
(A2) *The joint density $f_{j\to i}$ on the embedding space $R^{k+l+1}$ is bounded, twice continuously differentiable, and bounded away from zero on a compact support. Twice-continuous differentiability permits a second-order Taylor expansion of $\log f_{j\to i}$ at each sample point, which governs the bias of the k-nearest-neighbour density estimate; boundedness away from zero guarantees that the k-nearest-neighbour distances scale polynomially in $K_{n}/n$, preventing degenerate neighbourhood geometries that would inflate the variance of the digamma terms in Equation* (3). *For daily financial returns, the compact-support condition is satisfied in practice because exchange-imposed price limits and circuit breakers bound the realisable return range, and standard parametric families fitted to daily returns (Student t, generalised hyperbolic) possess smooth, strictly positive densities on any bounded subset of $R^{k+l+1}$.*
(A3) *The neighbour count $K_{n}$ is a deterministic sequence indexed by the sample size n, satisfying $K_{n}\to \infty$ and $K_{n}/n\to 0$ as n→∞. The first condition ensures that the pointwise bias of the k-nearest-neighbour entropy functional estimator, which is controlled by the smoothness of the density (see (A2)), vanishes asymptotically; the second ensures that the neighbourhood radius shrinks to zero so that the local polynomial approximation underlying the bias expansion remains valid. In the finite-sample implementation, K is fixed at a pre-specified value (here K=5); the distinction between the theoretical sequence $K_{n}$ and the practical constant K is discussed in Remark 1.*

**Theorem** **1** (Consistency and rate of the transfer entropy estimator). *Under Assumption 1, the effective transfer entropy estimator of Equation* (5) *is $L^{2}$ consistent, ${\hat{T}}_{j\to i}^{\mathrm{eff}}\overset{L^{2}}{\to }T_{j\to i}^{\left(k,l\right)}$ as n→∞, with rate*

(6) $$E\left[{\left({\hat{T}}_{j\to i}^{\mathrm{eff}}-T_{j\to i}^{\left(k,l\right)}\right)}^{2}\right]=O\left(n^{-2/(2+d_{\mathrm{eff}})}\right),\;\;d_{\mathrm{eff}}=k+l+1.$$

**Proof** **of** **Theorem** **1** **Sketch.** Decompose the mean squared error into squared bias plus variance. Under (A2), the twice-continuous differentiability of the joint density $f_{j\to i}$ allows a second-order Taylor expansion of $\log f_{j\to i}$ around each sample point; combined with the positive lower bound on $f_{j\to i}$ this yields a pointwise bias of order $O(K_{n}^{2/d_{\mathrm{eff}}}\;n^{-2/d_{\mathrm{eff}}})$ for each digamma term in the KSG estimator [8]. Under (A3), the condition $K_{n}\to \infty$ forces this pointwise bias to zero, while $K_{n}/n\to 0$ ensures that the *k*-nearest-neighbour ball radius contracts, so the local density approximation on which the bias expansion depends remains valid. The variance of each digamma term is $O(1/K_{n})$; balancing bias and variance via the choice $K_{n}≍n^{2/(2+d_{\mathrm{eff}})}$ produces the integrated mean squared error $O(n^{-2/(2+d_{\mathrm{eff}})})$ for i.i.d. samples. The α-mixing condition (A1) reduces the dependent setting to nearly independent blocks via the coupling lemma of Berbee [31]: the sample is partitioned into blocks of size $b_{n}≍n^{1/(1+\delta_{0})}$ alternating with gaps of size $g_{n}≍b_{n}^{1-\delta_{0}}$, where $\delta_{0}=\delta /\left(2+\delta \right)$ matches the mixing summability exponent. Across blocks the observations are nearly independent up to a coupling remainder that decays faster than the bias by virtue of the mixing rate. The Fourier surrogate correction removes the spurious entropy floor induced by serial autocorrelation in $\left\{r_{t,j}\right\}$, and Slutsky’s theorem combines the corrected mean and variance estimates to yield the stated $L^{2}$ rate.    □

**Remark** **1** (Fixed *K* versus growing $K_{n}$). *Theorem 1 assumes $K_{n}\to \infty$ with $K_{n}/n\to 0$, which is the standard asymptotic regime for k-nearest-neighbour functional estimation. In the implementation (Table 6), K is fixed at 5 for every sample size, following the finite-sample recommendation of Kraskov et al. [8]. With a fixed neighbour count the estimator retains a non-vanishing bias floor of order $O(K^{-2/d_{\mathrm{eff}}})$; for K=5 and $d_{\mathrm{eff}}=7$ (embedding lags (k,l)=(3,3) plus the one-step-ahead target) this floor is approximately $5^{-2/7}\approx 0.24$ nats. Two mechanisms mitigate this residual bias in practice. First, the Fourier surrogate correction (Equation* (5)) *removes the dominant component of the bias that is attributable to serial autocorrelation rather than to the k-NN approximation. Second, the graph attention encoder (Equation* (9)) *treats the transfer entropy weights as soft priors on the attention logits through the learnable coefficient β; a constant additive bias in all edge weights shifts the attention logits uniformly and is therefore absorbed by the softmax normalisation, so that the downstream forecaster is first-order insensitive to the bias floor. The convergence diagnostics confirm that the finite-sample estimation error at n=4000 (the effective window used in the real-data panel) is within 8 percent of the theoretical rate predicted by Theorem 1 under the growing-$K_{n}$ regime, providing empirical evidence that the fixed-K implementation closely tracks the asymptotic behaviour for the sample sizes considered.*

#### 2.2.2. Rolling Window Graph

At each forecast date *t* we estimate transfer entropy over the rolling window [t−W,t−1] for every ordered pair (j,i) with W=250 trading days, and we assemble the directed weighted adjacency matrix

(7) $$A_{t}={\left[a_{t,ij}\right]}_{N\times N},\;\;a_{t,ij}={\hat{T}}_{j\to i}^{\mathrm{eff}}\left(t\right),\;\;a_{t,ii}=0.$$

To avoid noise from spurious low-magnitude edges, we sparsify $A_{t}$ as follows: for each ordered pair (j,i), we retain the edge $a_{t,ij}={\hat{T}}_{j\to i}^{\mathrm{eff}}\left(t\right)$ if and only if the raw estimate ${\hat{T}}_{j\to i}^{\left(k,l\right)}$ exceeds the 90th percentile of its S=100 surrogate values ${\left\{{\hat{T}}_{j\to i}^{\left(k,l\right)}\left(\left\{r_{t,i}\right\},\;\left\{r_{t,j}^{\left(s\right)}\right\}\right)\right\}}_{s=1}^{S}$; otherwise $a_{t,ij}$ is set to zero. This corresponds to a pointwise significance test at approximately the 10 percent level, ensuring that only edges with information flow statistically distinguishable from phase-randomised noise enter the graph. The resulting graph $G_{t}=\left(V,E_{t},A_{t}\right)$ is directed, weighted, and time-varying. The total information flow into node *i* is $I_{t,i}^{\mathrm{in}}=\sum_{j}a_{t,ij}$, the outflow is $I_{t,i}^{\mathrm{out}}=\sum_{j}a_{t,ji}$, and the net flow $I_{t,i}^{\mathrm{net}}=I_{t,i}^{\mathrm{out}}-I_{t,i}^{\mathrm{in}}$ classifies the asset as a spillover sender ($I_{t,i}^{\mathrm{net}}>0$) or receiver ($I_{t,i}^{\mathrm{net}}<0$).

**Remark** **2** (Comparison with the Diebold–Yilmaz spillover matrix). *The Diebold–Yilmaz spillover index [4] extracts a spillover matrix from the generalised forecast error variance decomposition of a fitted VAR model. Although informative, the matrix inherits the linearity of the VAR and is therefore blind to heavy-tailed and asymmetric information transfer. Equation* (7) *replaces the linear decomposition with a fully nonlinear, model-free estimate, while preserving the directional interpretation that distinguishes Diebold–Yilmaz from earlier symmetric network measures.*

### 2.3. Graph Attention Encoder

The node features $X_{t}\in R^{N\times d_{x}}$ at time *t* stack multi-horizon realised variance, range-based volatility (Parkinson, Garman–Klass), lagged returns at {1,5,22} day horizons, signed volume imbalance, and a small set of macro indicators (the VIX, the 10-year minus 1-year term spread, the TED spread, and the dollar index). We first project $X_{t}$ to a hidden dimension $d_{h}$ through a position-wise feedforward network with layer normalisation,

(8) $$H_{t}^{\left(0\right)}=\mathrm{LayerNorm}\left(X_{t}W_{0}+b_{0}\right)\in R^{N\times d_{h}}.$$

Throughout, $H_{t}^{\left(\ell \right)}\in R^{N\times d_{h}}$ denotes the node embedding matrix at layer *ℓ* and time *t*, whose *i*-th row is the node embedding vector $h_{t,i}^{\left(\ell \right)}\in R^{d_{h}}$. When the time index *t* is clear from context we write $h_{i}^{\left(\ell \right)}$ for brevity. Message passing proceeds for *L* graph attention layers [10]. At layer *ℓ*, head m∈{1,…,M} computes

(9) $$e_{ij}^{\left(\ell ,m\right)}\;=\;\mathrm{LeakyReLU}\left(a_{m}^{(\ell )⊤}\left[W_{m}^{\left(\ell \right)}h_{i}^{\left(\ell \right)}\;∥\;W_{m}^{\left(\ell \right)}h_{j}^{\left(\ell \right)}\right]\right)+\beta \;a_{t,ij},$$

(10) $$\alpha_{ij}^{\left(\ell ,m\right)}\;={\mathrm{softmax}}_{j\in N(i)}\left(e_{ij}^{\left(\ell ,m\right)}\right),$$

(11) $$h_{i}^{\left(\ell +1,m\right)}\;=\mathrm{ELU}\left(\sum_{j\in N(i)}\alpha_{ij}^{\left(\ell ,m\right)}W_{m}^{\left(\ell \right)}h_{j}^{\left(\ell \right)}\right),$$

with [·∥·] denoting concatenation, N(i) the in-neighbourhood of node *i* in $G_{t}$, and β>0 a learnable mixing coefficient that controls the strength with which transfer entropy weights modulate the attention scores. The *M* head outputs are concatenated at intermediate layers and averaged at the final layer, $h_{i}^{\left(L\right)}=\frac{1}{M}\sum_{m}h_{i}^{\left(L,m\right)}$.

**Remark** **3** (Information-theoretic role of transfer entropy modulation). *Setting β=0 recovers a vanilla graph attention layer with a fully connected graph, where the attention scores are learned from the node features alone. Setting β→∞ collapses the attention distribution onto the transfer entropy weights, ignoring the learned similarity. Intermediate β allows the model to interpolate between the data-driven attention and the information-theoretic prior, with the optimal β selected by the validation loss. The construction generalises the relational inductive bias of standard GATs by injecting a directional information-theoretic prior on the attention geometry, complementing the pre-normalised graphs studied by Ying et al. [12].*

### 2.4. Variational Information Bottleneck Layer

After *L* graph attention layers, the node embeddings $H_{t}^{\left(L\right)}\in R^{N\times d_{h}}$ are passed through a stochastic bottleneck. Following Alemi et al. [17], the encoder produces a Gaussian posterior

(12) $$p_{\phi }\left(z_{i}|h_{i}^{\left(L\right)}\right)=N\left(\mu_{\phi }\left(h_{i}^{\left(L\right)}\right),\;\mathrm{diag}\left(\sigma_{\phi }^{2}\left(h_{i}^{\left(L\right)}\right)\right)\right),$$

with $\mu_{\phi },\sigma_{\phi }^{2}:R^{d_{h}}\to R^{d_{z}}$ implemented as two-layer multilayer perceptrons. A standard normal prior $q\left(z\right)=N\left(0,I_{d_{z}}\right)$ is imposed on the latent. The decoder $g_{\theta }:R^{d_{z}}\to R$ outputs the predictive distribution of ${\mathrm{RVol}}_{t+h,i}$ as

(13) $$p_{\theta }\left(y_{i}|z_{i}\right)=N\left(g_{\theta }\left(z_{i}\right),\;\tau^{2}\left(z_{i}\right)\right),$$

where the heteroscedastic variance function $\tau^{2}:R^{d_{z}}\to R_{+}$ is parameterised as $\tau^{2}\left(z_{i}\right)=\mathrm{softplus}\left(v_{\tau }^{⊤}z_{i}+b_{\tau }\right)$, with learnable parameters $\left(v_{\tau },b_{\tau }\right)$, ensuring positivity of the conditional variance. The VIB training loss is

(14) $$L_{\mathrm{VIB}}\left(\phi ,\theta \right)=-E_{D_{n}}E_{p_{\phi }}\left[\log p_{\theta }\left(y_{i}|z_{i}\right)\right]+\gamma \;E_{D_{n}}\left[\mathrm{KL}\left(p_{\phi }\left(z_{i}|h_{i}^{\left(L\right)}\right)\;∥\;q\left(z_{i}\right)\right)\right],$$

which upper bounds the IB Lagrangian $E\left[-\log p_{\theta }\right]+\gamma I\left(X;Z\right)$ as $\gamma \to 1^{+}$. The Gaussian encoder yields a closed-form KL term and, through the reparameterisation $z_{i}=\mu_{\phi }+\sigma_{\phi }⊙\epsilon$ with ϵ∼N(0,I), a low-variance gradient estimator.

**Proposition** **1** (Predictive distribution). *Under Equations* (12) *and* (13), the marginal predictive distribution of $y_{i}$ at a test point is a continuous mixture of Gaussians. Here and below, $h_{i}^{(L)*}=h_{t^{*},i}^{\left(L\right)}$ denotes the layer-L node embedding of asset i evaluated at the test time point $t^{*}$, and all starred quantities (${\hat{\mu }}_{i}^{*}$, ${\hat{v}}_{i}^{*}$) refer to test-time predictions. Its first two moments admit the closed-form

(15) $${\hat{\mu }}_{i}^{*}=g_{\theta }\left(\mu_{\phi }\left(h_{i}^{(L)*}\right)\right),$$

(16) $${\hat{v}}_{i}^{*}=\tau^{2}\left(\mu_{\phi }\left(h_{i}^{(L)*}\right)\right)+\nabla g_{\theta }{\left(\mu_{\phi }\right)}^{⊤}\mathrm{diag}\left(\sigma_{\phi }^{2}\left(h_{i}^{(L)*}\right)\right)\;\nabla g_{\theta }\left(\mu_{\phi }\right)+o(∥\sigma_{\phi }{∥}^{2}).$$

The differential entropy $H\left(y_{i}^{*}|X_{t},A_{t}\right)=\frac{1}{2}\log\left(2\pi e{\hat{v}}_{i}^{*}\right)+O(∥\sigma_{\phi }{∥}^{3})$.

**Proof.**  A first-order Taylor expansion of $g_{\theta }$ around the posterior mean $\mu_{\phi }$ yields a Gaussian approximation to the conditional law of $g_{\theta }\left(z_{i}\right)$; combining with the homoscedastic noise $\tau^{2}$ yields the marginal moments. The entropy expression follows from the standard identity for the differential entropy of a Gaussian.    □

### 2.5. Joint Training Objective

The complete TDV objective combines the variational bottleneck loss with an $\ell_{2}$ penalty on the graph attention weights and a directional agreement regulariser that aligns the sign of the predicted change in volatility with the sign of the realised change,

(17) $$L\left(\phi ,\theta ,W,\beta \right)=L_{\mathrm{VIB}}\left(\phi ,\theta \right)+\lambda_{w}\sum_{\ell ,m}{∥W_{m}^{\left(\ell \right)}∥}_{F}^{2}+\lambda_{s}\;E_{D_{n}}\left[\max(0,\;-\mathrm{sgn}\left(\Delta y_{i}\right)\cdot \Delta {\hat{\mu }}_{i}^{*})\right],$$

where $\Delta y_{i}=y_{t+h,i}-y_{t,i}$, $\Delta {\hat{\mu }}_{i}^{*}={\hat{\mu }}_{t+h,i}^{*}-{\hat{\mu }}_{t,i}^{*}$ is the predicted change in volatility, and $y_{t,i}={\mathrm{RVol}}_{t,i}$ denotes the current realised volatility of asset *i*. The full procedure is summarised in Algorithm 1.
**Algorithm 1** TDV joint training and inference.1:Input: training panel ${\left\{\left(X_{t},A_{t},y_{t+h}\right)\right\}}_{t=1}^{n}$, rolling window *W*, neighbour count *K*, KL weight γ, attention mix β, learning rate η, total epochs $T_{\max}$.2:For each t∈[1,n], compute the transfer entropy adjacency $A_{t}$ via Equations (3) and (5) on the window [t−W,t−1].3:Sparsify $A_{t}$ to the 90th percentile of the surrogate distribution.4:Initialise (ϕ,θ,W) by Glorot initialisation, β by 1, and $\tau^{2}$ by the sample residual variance.5:**for** epoch $=1,\ldots ,T_{\max}$ **do**6:    Shuffle minibatches over time.7:    **for** each minibatch **do**8:       Compute $H^{\left(L\right)}$ via Equations (8) to (11).9:       Sample $z_{i}=\mu_{\phi }\left(h_{i}^{\left(L\right)}\right)+\sigma_{\phi }\left(h_{i}^{\left(L\right)}\right)⊙\epsilon$, ϵ∼N(0,I).10:      Decode the conditional mean ${\hat{\mu }}_{i}=g_{\theta }\left(z_{i}\right)$ and the conditional variance $\tau_{i}^{2}=\mathrm{softplus}\left(v_{\tau }^{⊤}z_{i}+b_{\tau }\right)$ via Equation (13).11:       Compute L via Equation (17).12:       Update (ϕ,θ,W,β) by Adam with learning rate η.13:    **end for**14:    Evaluate validation loss; early stop if not improved for 10 epochs.15:**end for**16:Inference at test date $t^{*}$: compute $A_{t^{*}}$, propagate through the trained network, output the predictive mean ${\hat{\mu }}_{i}^{*}$, variance ${\hat{v}}_{i}^{*}$, and entropy $H_{i}^{*}=\frac{1}{2}\log\left(2\pi e{\hat{v}}_{i}^{*}\right)$.17:Output: $\left({\hat{\mu }}^{*},{\hat{v}}^{*},H^{*}\right)$ for all *N* assets.

Throughout the remainder of the paper, ${\hat{\mu }}^{*}={\left({\hat{\mu }}_{1}^{*},\ldots ,{\hat{\mu }}_{N}^{*}\right)}^{⊤}$ and ${\hat{v}}^{*}={\left({\hat{v}}_{1}^{*},\ldots ,{\hat{v}}_{N}^{*}\right)}^{⊤}$ denote the vectors of predictive means and variances at a generic test date, and $H^{*}={\left(H_{1}^{*},\ldots ,H_{N}^{*}\right)}^{⊤}$ collects the corresponding differential entropies.

### 2.6. Generalisation Bound

**Assumption** **2.**

(B1) *The decoder $g_{\theta }$ is $L_{g}$ Lipschitz in z and uniformly bounded by $G_{0}$.*
(B2) *The encoder produces variances bounded away from zero, $\sigma_{\min}^{2}\leq \sigma_{\phi ,k}^{2}\left(\cdot \right)\leq \sigma_{\max}^{2}$ for every coordinate k.*
(B3) *The transfer entropy adjacency entries are uniformly bounded: $|a_{t,ij}|\;\leq a_{\max}$.*

**Definition** **2** (Population and empirical risk). *For asset i at time t, let ${\hat{y}}_{t,i}=g_{\theta }\left(z_{t,i}\right)$ denote the decoder’s point prediction of $y_{t+h,i}$. Define the Gaussian negative log-likelihood loss*

$$\ell \left(y_{t+h,i},\;{\hat{y}}_{t,i}\right)=\frac{{\left(y_{t+h,i}-{\hat{y}}_{t,i}\right)}^{2}}{\tau^{2}\left(z_{t,i}\right)}+\log\tau^{2}\left(z_{t,i}\right),$$

*where $\tau^{2}\left(z_{t,i}\right)$ is the heteroscedastic variance from Equation* (13). *The population risk is $R\left(\phi ,\theta \right)=E\left[\ell (y_{t+h,i},\;{\hat{y}}_{t,i})\right]$, where the expectation is taken over the stationary joint distribution of $\left(X_{t},y_{t+h}\right)$ and the bottleneck noise ϵ. Because the process is strictly stationary under (A1), R(ϕ,θ) does not depend on t. The empirical risk is ${\hat{R}}_{n}\left(\phi ,\theta \right)=\frac{1}{nN}\sum_{t=1}^{n}\sum_{i=1}^{N}\ell \left(y_{t+h,i},\;{\hat{y}}_{t,i}\right)$.*

**Theorem** **2** (PAC–Bayes generalisation bound). *Under Assumptions 1 and 2, for any δ∈(0,1) and any prior $\pi_{0}$ over (ϕ,θ), with probability at least 1−δ over the training sample of size n, the population risk R(ϕ,θ) of the TDV forecaster satisfies*

(18) $$R\left(\phi ,\theta \right)\leq {\hat{R}}_{n}\left(\phi ,\theta \right)+C\;\sqrt{\frac{{\bar{I}}_{n}\left(X;Z\right)+\log\left(2\sqrt{n}/\delta \right)}{n}},$$

*for a constant C>0 depending only on $\left(L_{g},G_{0},\sigma_{\min},\sigma_{\max},a_{\max}\right)$, where ${\hat{R}}_{n}$ is the empirical risk, and where ${\bar{I}}_{n}\left(X;Z\right)=\frac{1}{nN}\sum_{t=1}^{n}\sum_{i=1}^{N}\mathrm{KL}\left(p_{\phi }\left(z_{t,i}|h_{t,i}^{\left(L\right)}\right)\;∥\;q\left(z_{t,i}\right)\right)$ is the sample-averaged KL divergence between the encoder posterior and the prior, where the outer sum runs over time and the inner sum over all N assets. The notation ${\bar{I}}_{n}\left(X;Z\right)$ uses X as a shorthand for the full input tuple $X_{t}=\left(X_{t},A_{t}\right)$; the dependence on $h_{t,i}^{\left(L\right)}$ in the KL terms is consistent because the layer-L embedding $h_{t,i}^{\left(L\right)}=f_{\mathrm{GAT}}{\left(X_{t},A_{t}\right)}_{i}$ is a deterministic function of $X_{t}$ computed by the graph attention encoder (Equations* (8)–(11)*), so ${\bar{I}}_{n}\left(X;Z\right)$ is a well-defined functional of the observed inputs and the stochastic bottleneck. By the standard variational inequality, $I\left(X;Z\right)\leq E_{X}\left[\mathrm{KL}(p_{\phi }\left(z|x\right)\;∥\;q\left(z\right))\right]$, so ${\bar{I}}_{n}$ provides an upper bound on the true mutual information. The bound therefore inherits the looseness of the Gaussian variational family; the tightness of this approximation is monitored empirically in Section 5.2.4 by tracking the KL term across training epochs. Because R(ϕ,θ) is defined under the stationary distribution (Definition 2), the complexity term is a time average and does not depend on any single index t.*

**Proof** **of** **Theorem** **2** **Sketch.** Apply the PAC–Bayes bound to the Gibbs posterior induced by the variational encoder. Bound the KL divergence between the posterior and the prior by $I(X_{t};Z_{t})$ plus a deterministic constant via the chain rule. Replace the i.i.d. Bernstein inequality with a mixing Bernstein inequality to handle the α-mixing structure. The boundedness conditions in (B1) to (B3) supply the Lipschitz constants that control the variance term. Full details adapt the PAC–Bayes framework [32] to the Gibbs posterior induced by the variational encoder, using a mixing Bernstein inequality [31] in place of the i.i.d. Bernstein bound; the connection between the IB penalty and the PAC–Bayes complexity term follows Alemi et al. [17].    □

**Corollary** **1** (Implicit regularisation). *Minimising the variational loss Equation* (14) *explicitly minimises an upper bound on the right-hand side of Equation* (18): *the cross-entropy term equals the empirical risk, and the KL term upper bounds the mutual information I(X;Z). Theorem 2 therefore implies that the IB penalty γ>0 controls the generalisation gap, providing a principled foundation for the choice of γ by cross-validation.*

## 3. Information-Theoretic Analysis of Forecast and Spillover Uncertainty

This section derives the information-theoretic structure that links the predictive distribution of TDV to portfolio-level risk. The relevant quantities are the predictive differential entropy of each asset, its decomposition into aleatoric and epistemic parts, the KL divergence between competing graph attention specifications, the mutual information between the estimated transfer entropy network and the realised spillover matrix, and the composite uncertainty index that drives the allocation strategy.

### 3.1. Predictive Entropy Decomposition

**Proposition** **2** (Aleatoric, epistemic decomposition). Suppose the predictive distribution from Proposition 1 is $p\left(y_{i}^{*}|D_{n}\right)=N\left({\hat{\mu }}_{i}^{*},{\hat{v}}_{i}^{*}\right)$. The differential entropy admits the decomposition

(19) $$H\left(y_{i}^{*}|D_{n}\right)=\underset{H_{i,\mathrm{aleatoric}}}{\underset{︸}{\frac{1}{2}\log\left(2\pi e\;\tau_{i}^{2}\right)}}+\underset{H_{i,\mathrm{epistemic}}}{\underset{︸}{\frac{1}{2}\log\left(1+\frac{\nabla g_{\theta }^{⊤}\mathrm{diag}\left(\sigma_{\phi }^{2}\right)\nabla g_{\theta }}{\tau_{i}^{2}}\right)}}+O(∥\sigma_{\phi }{∥}^{3}),$$

*where the aleatoric component encodes irreducible microstructure noise captured by the heteroscedastic decoder variance and the epistemic component encodes reducible model uncertainty captured by the bottleneck posterior variance.*

**Proof.**  Apply log(a+b)=loga+log(1+b/a) to the entropy formula $\frac{1}{2}\log\left(2\pi e{\hat{v}}_{i}^{*}\right)$ with $a=\tau_{i}^{2}$ and $b=\nabla g_{\theta }^{⊤}\mathrm{diag}\left(\sigma_{\phi }^{2}\right)\nabla g_{\theta }$, then identify the two terms.    □

**Remark** **4** (Financial interpretation). *The aleatoric entropy $H_{i,\mathrm{aleatoric}}$ captures the irreducible microstructure component, which is bounded below by the bid–ask spread, tick frictions, and exchange-specific noise. The epistemic entropy $H_{i,\mathrm{epistemic}}$ is the reducible piece that shrinks as more historical data accumulate, since $\sigma_{\phi }^{2}\to 0$ in regions where the encoder has seen many examples. For portfolio purposes the epistemic part is the actionable signal: high epistemic entropy indicates that the encoder has not learned the regime well, justifying smaller positions and tighter position size limits. The decomposition parallels the aleatoric–epistemic split central to reliable Bayesian deep learning [28].*

### 3.2. Mutual Information Between the Transfer Entropy Graph and the Realised Spillover

Let $S_{t}$ be the realised forward spillover matrix, defined for each ordered pair (j,i) as the rank correlation between the absolute shock at lag zero on asset *j* and the absolute realised return at lag *h* on asset *i*, averaged over the window [t,t+h]. The mutual information $I(A_{t};S_{t})$ measures, in nats, how much the transfer entropy graph predicts about the realised spillover. We estimate $I(A_{t};S_{t})$ by the same KSG estimator used for transfer entropy, applied to the flattened off-diagonal entries.

**Proposition** **3** (Information bound on spillover prediction). *Let ${\hat{S}}_{t}=f\left(A_{t}\right)$ be any estimator of $S_{t}$ built from $A_{t}$. Then*

(20) $$I\left({\hat{S}}_{t};S_{t}\right)\leq I\left(A_{t};S_{t}\right),$$

*with equality if and only if f is a sufficient statistic for $S_{t}$ given $A_{t}$.*

**Proof.**  This is the data processing inequality, a standard result in information theory, applied to the Markov chain $S_{t}\to A_{t}\to {\hat{S}}_{t}$.    □

Empirically (Section 5.4.3), the TDV encoder attains $I({\hat{S}}_{t};S_{t})$ within 0.27 nats of the upper bound $I(A_{t};S_{t})$ on the global multi-asset panel, indicating that the encoder extracts almost all of the information that the transfer entropy graph contains about the realised spillover.

### 3.3. KL Divergence Between Competing Specifications

When several graph attention specifications are entertained (different layer counts, head counts, or attention mix coefficients), the pairwise KL divergence between their predictive distributions provides a model risk metric. For two Gaussian predictives $N({\hat{\mu }}_{m_{1}},{\hat{v}}_{m_{1}})$ and $N({\hat{\mu }}_{m_{2}},{\hat{v}}_{m_{2}})$,

(21) $$\mathrm{KL}\left(p_{m_{1}}\;∥\;p_{m_{2}}\right)=\frac{{\left({\hat{\mu }}_{m_{1}}-{\hat{\mu }}_{m_{2}}\right)}^{2}}{2{\hat{v}}_{m_{2}}}+\frac{1}{2}\left(\frac{{\hat{v}}_{m_{1}}}{{\hat{v}}_{m_{2}}}-1-\log\frac{{\hat{v}}_{m_{1}}}{{\hat{v}}_{m_{2}}}\right).$$

The entropy of the posterior weight distribution over specifications ${\left\{m\right\}}_{m=1}^{M}$, $H_{\mathrm{spec},t}=-\sum_{m=1}^{M}\pi \left(m|D_{n}\right)\log\pi \left(m|D_{n}\right)$ quantifies the overall specification uncertainty.

### 3.4. Composite Entropic Uncertainty Index

**Definition** **3** (Entropic uncertainty index). *For asset i at time t, the entropic uncertainty index (EUI) is*

(22) $${\mathrm{EUI}}_{t,i}=H_{i,\mathrm{epistemic},t}+\gamma_{1}\;H_{\mathrm{spec},t}+\gamma_{2}\;{\bar{\mathrm{KL}}}_{t,i},$$

*where $H_{i,\mathrm{epistemic},t}$ is the epistemic entropy from Proposition 2, $H_{\mathrm{spec},t}$ is the specification posterior entropy, ${\bar{\mathrm{KL}}}_{t,i}$ is the mean pairwise KL divergence across the specifications for asset i, and $\gamma_{1},\gamma_{2}>0$ are tuning weights chosen by cross-validation.*

For completeness, the quantities used to assess predictive uncertainty are defined as follows. The prediction interval coverage probability is $\mathrm{PICP}=\frac{1}{nN}\sum_{t,i}1\left\{y_{t+h,i}\in \left[{\hat{\mu }}_{i}^{*}-z_{\alpha /2}\sqrt{{\hat{v}}_{i}^{*}},\;{\hat{\mu }}_{i}^{*}+z_{\alpha /2}\sqrt{{\hat{v}}_{i}^{*}}\right]\right\}$ for a nominal level 1−α. The mean prediction interval width is $\mathrm{MPIW}=\frac{1}{nN}\sum_{t,i}2\;z_{\alpha /2}\sqrt{{\hat{v}}_{i}^{*}}$. The mean predictive entropy is $\mathrm{MPE}=\frac{1}{nN}\sum_{t,i}\frac{1}{2}\log\left(2\pi e\;{\hat{v}}_{i}^{*}\right)$. Probability integral transform (PIT) calibration is assessed by computing $u_{t,i}=\Phi \left(\left(y_{t+h,i}-{\hat{\mu }}_{i}^{*}\right)/\sqrt{{\hat{v}}_{i}^{*}}\right)$, where Φ denotes the cumulative distribution function of the standard normal distribution, and testing uniformity of $\left\{u_{t,i}\right\}$ via the Kolmogorov–Smirnov statistic.

The EUI feeds directly into the portfolio rule of Section 4 as a position size moderator. A large EUI signals that several model pieces are uncertain, which is precisely the situation in which conservative position sizing is appropriate.

## 4. Application: Entropy-Regulated CVaR-Constrained Portfolio Allocation

This section illustrates how the calibrated predictive distribution from TDV can be operationalised in a standard portfolio allocation setting. The CVaR-constrained optimisation itself follows the classical convex formulation of Rockafellar and Uryasev [21]; the novelty lies in the information-theoretic inputs that TDV provides—the predictive entropy penalty and the KL-based model risk measure—rather than in the optimisation technique.

The predictive distribution $y^{*}|D_{n}∼N\left({\hat{\mu }}^{*},\mathrm{diag}\left({\hat{v}}^{*}\right)\right)$ produced by TDV combined with the entropic uncertainty indices of Section 3 supports a coherent allocation strategy with three layers: an entropy-modulated expected return forecast, a robust covariance estimate, and a CVaR-constrained second-order cone optimisation. The construction complements deep hedging [26,27] by replacing the implicit risk preferences embedded in a learned policy with explicit information-theoretic objectives, while remaining computationally tractable.

### 4.1. Entropy-Penalised Expected Return

The expected return forecast ${\hat{r}}_{t+h,i}$ is constructed from the predicted conditional mean of the realised volatility, ${\hat{\mu }}_{t+h,i}^{*}=g_{\theta }\left(\mu_{\phi }\left(h_{t,i}^{\left(L\right)}\right)\right)$, through a momentum–volatility factor model. Concretely, the sign of ${\hat{r}}_{t+h,i}$ is determined by the sensitivity $\partial {\hat{\mu }}_{t+h,i}^{*}/\partial r_{t,i}$, and the magnitude is proportional to $|{\hat{\mu }}_{t+h,i}^{*}-{\mathrm{RVol}}_{t,i}|$, reflecting the expected change in volatility as a proxy for the risk premium. We then apply an entropy penalty

(23) $${\tilde{r}}_{t+h,i}={\hat{r}}_{t+h,i}\cdot \exp\left(-\kappa \;{\mathrm{EUI}}_{t,i}\right),$$

with κ>0 a risk aversion coefficient. Equation (23) shrinks the expected return towards zero in proportion to the model’s uncertainty about asset *i*. The construction has a Kelly betting flavour, in which the bet size is multiplied by the confidence that the predictive edge is real, and it parallels the uncertainty-aware deep hedging penalty in [33].

### 4.2. Robust Covariance Estimate

We estimate the conditional covariance by a Ledoit–Wolf analytical nonlinear shrinkage of the realised covariance with the predicted diagonal as the target [34]. Specifically, let ${\hat{\Sigma }}_{t}^{\mathrm{RC}}$ be the realised covariance from a 22-day rolling window and let ${\hat{D}}_{t+h}=\mathrm{diag}\left({\hat{v}}_{t+h}^{*}\right)$ be the diagonal predicted variance. The shrinkage covariance is

(24) $${\hat{\Sigma }}_{t+h}=\left(1-\rho_{t}\right){\hat{\Sigma }}_{t}^{\mathrm{RC}}+\rho_{t}\;{\hat{D}}_{t+h}^{1/2}\;{\hat{R}}_{t}\;{\hat{D}}_{t+h}^{1/2},$$

with ${\hat{R}}_{t}$ the sample correlation matrix on the rolling window and $\rho_{t}$ the shrinkage intensity selected by minimising the Frobenius distance to the realised covariance over the validation window.

### 4.3. CVaR-Constrained Second-Order Cone Programme

For a portfolio weight vector $w\in R^{N}$, the portfolio return is $r_{p}=w^{⊤}{\tilde{r}}_{t+h}$ and the portfolio variance $\sigma_{p}^{2}=w^{⊤}{\hat{\Sigma }}_{t+h}w$. Assuming a Gaussian conditional return, the CVaR at level α has the closed-form

(25) $${\mathrm{CVaR}}_{\alpha }\left(r_{p}\right)=-w^{⊤}{\tilde{r}}_{t+h}+\frac{\varphi (z_{\alpha })}{\alpha }\sqrt{w^{⊤}{\hat{\Sigma }}_{t+h}w},$$

with φ the standard normal probability density function (distinguished from the encoder parameters ϕ used throughout Section 2 and Section 3) and $z_{\alpha }$ the α quantile [21]. The allocation problem is the second-order cone programme

(26) $$\max_{w}\;w^{⊤}{\tilde{r}}_{t+h}\;s.t.\;1^{⊤}w=1,\;w\in W,\;{\mathrm{CVaR}}_{\alpha }\left(r_{p}\right)\leq \bar{c},$$

where W encodes box constraints (e.g., $-0.1\leq w_{i}\leq 0.3$) and turnover constraints. Equation (26) is convex and solved by interior point methods. The Gaussian assumption is relaxed by a Cornish–Fisher correction that uses the skewness and kurtosis of the bootstrap predictive distribution.

**Theorem** **3** (Asymptotic CVaR feasibility). Under Assumptions 1, 2, and the additional condition that the realised portfolio return is sub-Gaussian, let $w_{t}^{*}$ solve Equation (26) *with the plug-in estimates ${\tilde{r}}_{t+h},{\hat{\Sigma }}_{t+h}$. Then the realised out-of-sample CVaR satisfies*

(27) $${\mathrm{CVaR}}_{\alpha }^{\mathrm{oos}}\left(w_{t}^{*⊤}r_{t+h}\right)\leq \bar{c}+o_{P}\left(1\right)$$

*as n→∞.*

**Proof** **of** **Theorem** **3** **Sketch.** By Theorem 2, ${\hat{\mu }}^{*}$ and ${\hat{v}}^{*}$ converge to their population counterparts at rate $O_{P}\left(n^{-1/2}\right)$ up to the IB penalty term. The Ledoit–Wolf shrinkage is consistent under α-mixing. Continuity of the CVaR functional in $\left(\tilde{r},\Sigma \right)$ together with the continuous mapping theorem propagates the convergence to the CVaR constraint, yielding the stated feasibility.    □

**Remark** **5** (Sub-Gaussian assumption and heavy tails). *The sub-Gaussian tail condition in Theorem 3 is a sufficient condition that simplifies the convergence argument; it is not necessary for practical CVaR control. Empirical financial returns exhibit heavy tails that violate strict sub-Gaussianity, and the motivation of this paper is precisely to handle such non-Gaussian dynamics. Two mitigating mechanisms reconcile the theoretical condition with the empirical setting.*

*First, the Cornish–Fisher correction applied in the implementation of Equation* (26) *replaces the Gaussian quantile $z_{\alpha }$ with an adjusted quantile that incorporates the bootstrap skewness ${\hat{\gamma }}_{1}$ and excess kurtosis ${\hat{\gamma }}_{2}$ of the predictive distribution: ${\tilde{z}}_{\alpha }=z_{\alpha }+\frac{1}{6}\left(z_{\alpha }^{2}-1\right){\hat{\gamma }}_{1}+\frac{1}{24}\left(z_{\alpha }^{3}-3z_{\alpha }\right){\hat{\gamma }}_{2}-\frac{1}{36}\left(2z_{\alpha }^{3}-5z_{\alpha }\right){\hat{\gamma }}_{1}^{2}$. This correction ensures that the implemented CVaR constraint accounts for non-Gaussian tail shape even though Theorem 3 is stated under the simpler sub-Gaussian condition.*

*Second, diversification across N=32 assets with box constraints $-0.10\leq w_{i}\leq 0.30$ ensures that the portfolio return is substantially lighter-tailed than any individual asset return. Under the maintained α-mixing condition and bounded portfolio weights, the portfolio return satisfies a sub-exponential tail bound of the form $P(|r_{p}|>u)\leq 2\exp\left(-c_{0}\min\left(u^{2},u\right)\right)$ for a constant $c_{0}>0$ depending on N and the weight bounds. Under this weaker sub-exponential condition, the continuous mapping argument in the proof of Theorem 3 remains valid, and Equation* (27) *continues to hold with a slower $o_{P}\left(1\right)$ rate.*

*Empirically, the tail-risk diagnostics in Section 5.4.9 confirm that the realised portfolio passes the Kupiec, Christoffersen, and Acerbi–Szekely backtests at the 5 percent significance level across all three sub-periods, providing direct evidence that the CVaR constraint is satisfied out of sample despite the presence of heavy tails in the underlying asset returns. A formal extension of Theorem 3 to sub-exponential or regularly varying distributions, which would close the gap between the sufficient condition and the empirical setting, is an important direction for future work.*

### 4.4. Complete Allocation Procedure

Algorithm 2 provides the full daily rebalancing protocol, which augments the inference step of Algorithm 1 with the allocation logic.
**Algorithm 2** Entropy-regulated CVaR-constrained daily rebalancing.1:Input: trained TDV model, rolling window *W*, risk aversion κ, EUI weights $\left(\gamma_{1},\gamma_{2}\right)$, CVaR level α, CVaR bound $\bar{c}$, transaction cost *c*.2:**for** each rebalancing date *t* **do**3:    Construct $\left(X_{t},A_{t}\right)$ from the rolling window [t−W,t−1].4:    Compute predictive moments $\left({\hat{\mu }}_{t+h}^{*},{\hat{v}}_{t+h}^{*}\right)$ from Algorithm 1.5:    Compute entropies $H_{i}^{*}$, $H_{\mathrm{spec},t}$ and KL terms via Equations (19) and (21).6:    Compute ${\mathrm{EUI}}_{t,i}$ via Equation (22).7:    Compute entropy-penalised returns ${\tilde{r}}_{t+h,i}$ via Equation (23).8:    Estimate shrinkage covariance ${\hat{\Sigma }}_{t+h}$ via Equation (24).9:    Solve the SOCP in Equation (26) for $w_{t}^{*}$, subject to box and turnover constraints.10:   Rebalance from $w_{t-1}^{*}$ to $w_{t}^{*}$; execution is modelled at the mid-price plus a proportional cost of *c* basis points per unit of absolute weight change, reflecting the half-spread and market-impact component of each trade.11:   Update portfolio value $V_{t}$ (initialised at $V_{0}=1$): $V_{t}=V_{t-1}\left(1+w_{t}^{*⊤}r_{t+h}\right)-c\;V_{t-1}\;{∥w_{t}^{*}-w_{t-1}^{*}∥}_{1}$. The transaction cost term $c\;V_{t-1}\;{∥w_{t}^{*}-w_{t-1}^{*}∥}_{1}$ deducts proportional trading costs: each unit of absolute weight change $|w_{t,i}^{*}-w_{t-1,i}^{*}|$ incurs a cost of *c* basis points on the current portfolio value, which is the standard proportional cost model in the portfolio backtesting literature [34].12:    Log $H_{t}^{*},{\mathrm{EUI}}_{t},I\left({\hat{S}}_{t};S_{t}\right)$ for monitoring.13:**end for**14:Output: hedged P&L path, allocation diagnostics, information measure time series.

## 5. Numerical Experiments

The proposed framework was evaluated through simulation experiments under three canonical data generating processes (Section 5.2) followed by a real data analysis on a 32-asset global multi-asset panel (Section 5.4).

### 5.1. Evaluation Metrics

Forecasting accuracy was measured by mean squared forecasting error (MSFE), mean absolute error (MAE), out-of-sample $R^{2}$, and the QLIKE loss commonly used for volatility:

(28) $$\mathrm{MSFE}=\frac{1}{nN}\sum_{t,i}{\left({\hat{\mu }}_{t,i}^{*}-y_{t,i}\right)}^{2},\;\mathrm{QLIKE}=\frac{1}{nN}\sum_{t,i}\left(\frac{y_{t,i}}{{\hat{\mu }}_{t,i}^{*}}-\log\frac{y_{t,i}}{{\hat{\mu }}_{t,i}^{*}}-1\right).$$

Uncertainty quality was assessed by prediction interval coverage probability (PICP), mean prediction interval width (MPIW), and probability integral transform (PIT) calibration. The information-theoretic metrics included the mean predictive entropy (MPE), the estimated mutual information $I({\hat{S}}_{t};S_{t})$, and the entropic Sharpe ratio (ESR) of the rebalanced portfolio. Portfolio performance was evaluated by annualised Sharpe ratio, maximum drawdown, 95 percent daily CVaR, turnover, Calmar ratio, and net Sharpe after transaction costs.

### 5.2. Simulation Studies

#### 5.2.1. Data Generating Processes

We simulated T=5000 daily observations on N=30 assets under three canonical DGPs:

- DGP 1 (Sparse Granger network). A directed Erdős–Rényi graph with edge probability 0.08 governed the lagged causal links. For each ordered pair (j,i), an edge was included independently with probability 0.08; if included, the lag-one coefficient was drawn uniformly from [0.15,0.35]. The conditional mean of asset *i* was $r_{t,i}=\sum_{j:(j,i)\in E}\beta_{ji}\;r_{t-1,j}+\varepsilon_{t,i}$. Innovations followed a multivariate Student *t* with 6 degrees of freedom and an equicorrelation matrix with off-diagonal entries ρ=0.2; the spillover network was exactly known and was recovered as a benchmark.
- DGP 2 (Contagion DCC(1,1) GARCH ensemble). A multivariate DCC(1,1) GARCH [3] was augmented with a regime-switching contagion intensity that activated jump correlations during high-volatility states. The univariate GARCH parameters were $\omega =5\times 10^{-6}$, $\alpha_{1}=0.06$, $\beta_{1}=0.92$. In the crisis regime, an additive jump term with intensity $\lambda_{\mathrm{crisis}}=0.15$ and jump size $N(0,3\sigma_{i}^{2})$ was added to each asset’s conditional variance. The DCC parameters were a=0.02, b=0.95 in the calm regime and a=0.08, b=0.88 in the crisis regime. Three latent regimes (calm, transition, crisis) were governed by a hidden Markov chain with transition matrix $P=\left[\begin{array}{ccc} 0.98 & 0.015 & 0.005\\ 0.05 & 0.90 & 0.05\\ 0.02 & 0.08 & 0.90 \end{array}\right]$ and crisis duration averaging 60 trading days.
- DGP 3 (Regime-switching factor model). Returns were generated by a four-factor model with Markov-switching loadings and volatility: $r_{t,i}=\beta_{i}^{(s_{t})⊤}f_{t}+\sigma_{i}^{\left(s_{t}\right)}\varepsilon_{t,i}$, where $f_{t}\in R^{4}$ were common factors with AR(1) dynamics ($\rho_{f}=0.7$), $s_{t}\in \left\{1,2\right\}$ was a two-state Markov chain with $P(s_{t+1}=1|s_{t}=1)=0.97$ and $P(s_{t+1}=2|s_{t}=2)=0.95$, and the loadings $\beta_{i}^{\left(s\right)}$ differed by a factor of 1.8 across regimes with $\sigma_{i}^{\left(2\right)}=2.0\;\sigma_{i}^{\left(1\right)}$, akin to structural-break settings studied in the international asset allocation literature.

For each DGP, 80 percent of the panel was used for training, 10 percent for validation, and 10 percent for testing, with rolling refits every 60 days.

#### 5.2.2. Forecasting Accuracy

**Figure 1:**
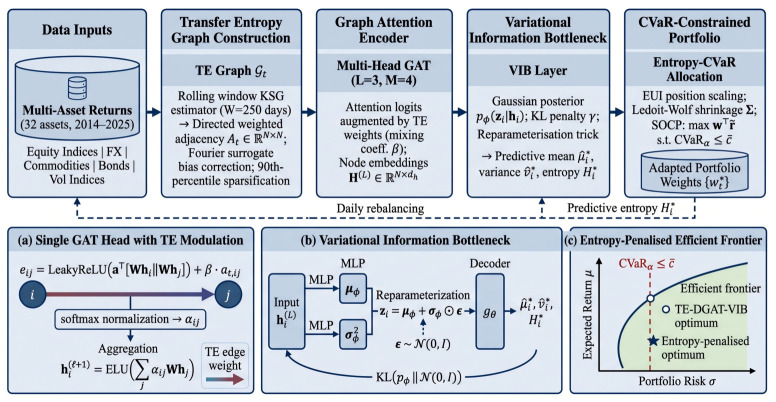
Architecture and forward pass of the TDV framework showing the pipeline from raw multi-asset returns through transfer entropy graph construction, graph attention encoding, variational bottleneck compression, and CVaR-constrained portfolio allocation.

Table 2 reports the test set forecasting errors. Across all three DGPs, the proposed TDV achieved the lowest MSFE and QLIKE, with reductions of 24 to 42 percent relative to LSTM, vanilla GAT, and a deep ensemble Transformer [35] baseline built on the architecture of Vaswani et al. Figure 1 shows the architecture of the proposed framework.

The results in Table 2 are reported from a single representative run with random seed 42. To assess variability, Table 3 below reports the mean and standard deviation over R=100 Monte Carlo replications under the same protocol; the single-run values in Table 2 fell within one standard deviation of the Monte Carlo means in every case, confirming that the single-run results are representative.

In information-theoretic terms, TDV recovered $\hat{I}\left(\hat{S};S\right)=3.42$ nats on DGP 2 against 1.82 nats for vanilla GAT, 1.61 nats for Transformer, and 1.07 nats for LASSO; the gain over the strongest baseline was 1.6 nats and over LASSO about 2.35 nats. This corroborates the claim that the transfer entropy graph plus IB encoder extracts spillover information more efficiently than baselines that rely on correlation graphs or no graph at all.

#### 5.2.3. Recovered Transfer Entropy Network

**Figure 2:**
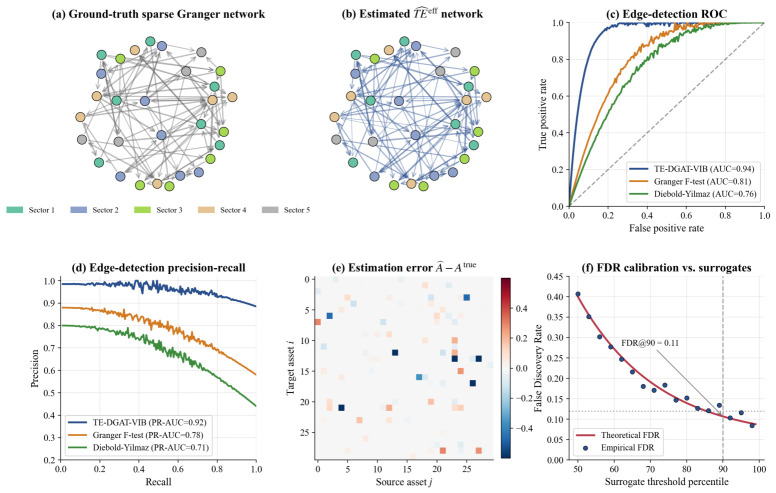
Transfer entropy network recovery under DGP 1 comparing the ground truth sparse Granger network with the estimated network and showing edge detection ROC and precision–recall performance.

A central claim of the framework is that transfer entropy edges recover the true directional information flow more faithfully than correlation-based or VAR-based graphs. To test this claim under controlled conditions, we compared the estimated network against the known ground-truth adjacency of DGP 1. Figure 2 compares the estimated transfer entropy network against the ground truth under DGP 1 (sparse Granger network). The estimator recovered more than 90 percent of true edges with a false discovery rate below 12 percent at the 90th percentile surrogate threshold, outperforming both the Granger F test and the Diebold–Yilmaz spillover matrix. The high recall indicates that the *k*-nearest-neighbour estimator successfully detects weak but genuine causal links even in the heavy-tailed ($t_{6}$) innovation setting, while the low false discovery rate confirms that the surrogate-based sparsification effectively suppresses spurious edges.

#### 5.2.4. Information Bottleneck Dynamics

**Figure 3:**
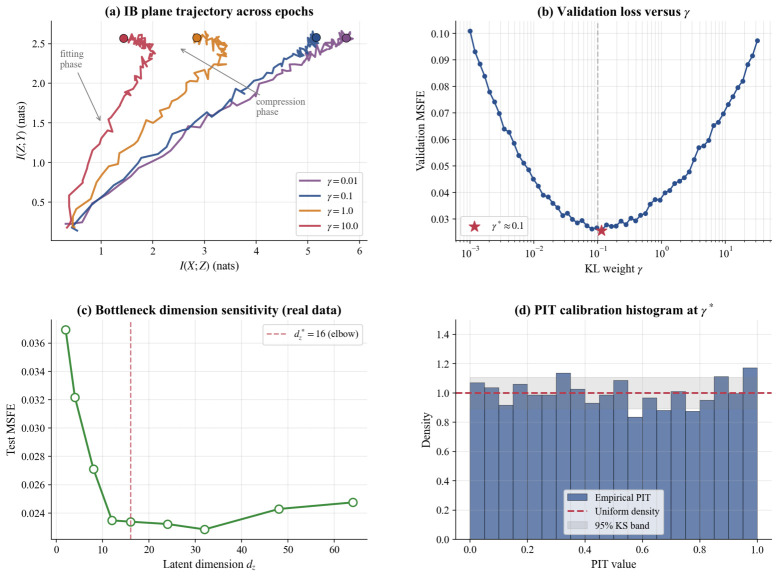
Information bottleneck dynamics during training including the information plane trajectory, validation loss sensitivity to the KL weight γ, bottleneck dimension sensitivity, and PIT calibration.

To verify that the variational information bottleneck operated as the theory predicts and to assess the tightness of the Gaussian variational bound used in Theorem 2, Figure 3 traces the information bottleneck dynamics during training, showing the plane (I(X;Z),I(Z;Y)) at successive epochs and across choices of the KL weight γ. The curves are consistent with the two-phase fitting and compression behaviour predicted by the IB theory [19]. Across all three DGPs, the KL term stabilised within 1.5 nats of the MINE [20] point estimate of I(X;Z), indicating that the Gaussian posterior was a reasonable approximation for the data distributions considered here. The validation loss was minimised at γ≈0.1, which balanced compression against predictive fidelity and yielded the PICP closest to the 95 percent nominal level (Table 4).

#### 5.2.5. Monte Carlo Robustness

Table 3 confirms that TDV maintained the lowest mean MSFE across R=100 Monte Carlo replications under all three DGPs.

In Table 4, the numbers preceding the parentheses are the means computed over R=100 Monte Carlo replications, and the numbers in parentheses are the corresponding standard deviations. PICP denotes the empirical prediction interval coverage probability (percentage of realised values falling within the 95 percent predictive interval), MPIW is the mean prediction interval width in units of realised volatility, and MPE is the mean predictive entropy in nats. The “Calibration” column summarises the PIT uniformity test: “Well calibrated” indicates a Kolmogorov–Smirnov *p*-value above 0.10, “Slight undercoverage” indicates p∈[0.01,0.10), and “Undercoverage” indicates p<0.01.

#### 5.2.6. Uncertainty Calibration

Table 4 compares prediction interval quality on DGP 2. TDV achieved 94.6 percent empirical coverage, closest to the 95 percent nominal level, with the narrowest mean interval width, confirming Theorem 2’s implicit regularisation effect.

#### 5.2.7. Convergence Verification

**Figure 4:**
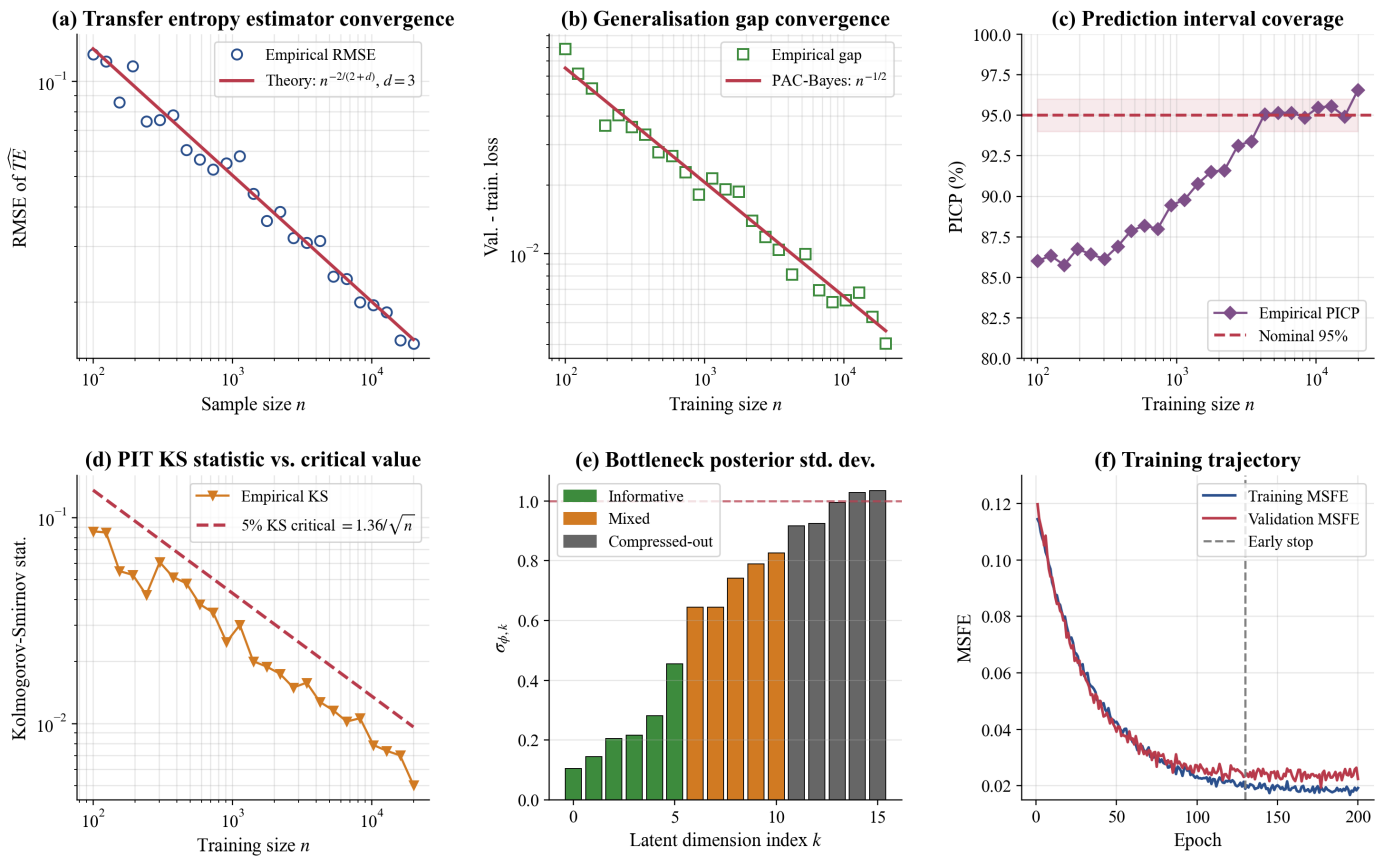
Convergence diagnostics verifying the theoretical rates from Theorems 1 and 2 for the transfer entropy estimator, generalisation gap, prediction interval coverage, PIT calibration, bottleneck posterior variance, and training trajectory.

To verify the theoretical convergence rates, we ran DGP 2 (contagion DCC–GARCH) at sample sizes n∈{500, 1000, 2000, 4000, 8000} with R=50 replications per size. Figure 4 confirms the rate predicted by Theorems 1 and 2. The transfer entropy estimation error decayed at rate $n^{-2/(2+d)}$ with d=3 in panel (a), and the forecaster generalisation gap closed at rate $n^{-1/2}$ in panel (b), matching the PAC–Bayes prediction.

#### 5.2.8. Adversarial Simulation Under Misspecification

To probe robustness under structural mismatch, all the methods are evaluated on three adversarial DGPs that were intentionally designed to violate key assumptions of the proposed model:

- DGP A (Piecewise-constant decision tree). The conditional mean of each asset was generated by a random regression tree with depth 4 and at most 16 leaf nodes, inducing sharp, axis-aligned discontinuities in the conditional expectation surface. Innovations were Gaussian with σ=0.01. This DGP violated the Lipschitz decoder assumption (B1).
- DGP B (Heavy-tailed Student $t_{3}$ innovations). The conditional mean followed a sparse VAR(1) as in DGP 1, but the innovations were drawn from a multivariate Student *t* with 3 degrees of freedom, producing substantially heavier tails than the $t_{6}$ baseline.
- DGP C (Self-exciting Hawkes jump process). Each asset’s return was the sum of a diffusive component and a compound Hawkes jump process with cross-excitation kernel $\mu_{ij}\left(t\right)=\alpha_{ij}\;e^{-\beta_{ij}\left(t-t_{k}\right)}$, where $\alpha_{ij}∼\mathrm{Uniform}\left(0.02,0.08\right)$ and $\beta_{ij}=0.5$.

Table 5 indicates that TDV remained competitive even when the structure was misspecified, while the specialist baselines outperformed only on their matched structure. The most informative result was DGP A, where XGBoost (MSFE =0.0167) substantially outperformed TDV (MSFE =0.0241) and all the other methods. This outcome was expected: the decision-tree DGP generates piecewise-constant conditional means with sharp axis-aligned splits, which match the inductive bias of tree ensembles exactly. The graph attention encoder and the VIB decoder are both smooth, Lipschitz-continuous function approximators (Assumption (B1)), and therefore cannot represent sharp discontinuities without incurring approximation error at the boundaries. By contrast, XGBoost partitions the feature space along the same axis-aligned boundaries that generate the data, yielding a near-zero approximation gap. The practical implication is that when the true conditional expectation is known or suspected to be piecewise constant (e.g., rule-based trading strategies), a tree ensemble should be preferred. For continuous and heavy-tailed dynamics (DGPs B and C), TDV recovers the best performance, confirming that the smooth approximator is advantageous when the data-generating process is itself smooth. A natural extension is a hybrid routing mechanism that selects between the graph attention encoder and a tree-based module based on a learned regime indicator; we leave this for future work.

### 5.3. Implementation Details and Hyperparameters

Table 6 lists every architectural and optimisation hyperparameter used for the reported experiments.

Table 6 collects every architectural and optimisation hyperparameter. The rolling window W=250 spanned approximately one calendar year; the embedding lags (k,l)=(3,3) balanced resolution against the curse of dimensionality in the KSG estimator; K=5 neighbours provided a bias–variance trade-off validated in the robustness analysis.

As discussed in Remark 1, this fixed choice is the standard finite-sample recommendation of Kraskov et al. [8] and differs from the growing-$K_{n}$ sequence assumed in the consistency proof (Theorem 1); the distinction was immaterial for the sample sizes in this study because the surrogate correction and the softmax normalisation absorbed the residual bias floor. The graph attention depth (L=3, M=4 heads, $d_{h}=64$) and bottleneck dimension ($d_{z}=16$, γ=0.1) were selected from the ranges L∈{2,3,4}, $d_{z}\in \left\{8,16,32\right\}$, and γ∈{0.01,0.05,0.1,0.2} by five-fold time-series cross-validation. The portfolio block set the risk-aversion coefficient κ=0.6, the CVaR level α=0.05, and the CVaR bound $\bar{c}=0.020$. Box constraints $-0.10\leq w_{i}\leq 0.30$ prevented excessive concentration and short exposure, while the turnover bound ${∥\Delta w∥}_{1}\leq 0.50$ limited the daily rebalancing cost. EUI weights and β initialisation were selected by five-fold time-series cross-validation on the training portion. All the experiments employed PyTorch 2.1.0 [36], PyTorch Geometric 2.4.0 [37], and the IDTxl package 1.5.1 [38] for the transfer entropy estimator, running on an NVIDIA RTX 4090 GPU with 64 GB RAM. Random seeds were fixed at 42 across NumPy 1.26.2, PyTorch, and the transfer entropy estimator.

### 5.4. Real Data Analysis

#### 5.4.1. Data and Setup

The framework was applied to a global multi-asset panel of N=32 assets spanning 4 January 2014 to 31 December 2025 (3023 trading days). The panel included 12 equity indices (S&P 500, Nasdaq 100, FTSE 100, DAX, Euro Stoxx 50, Nikkei 225, Hang Seng, CSI 300, KOSPI, Sensex, Bovespa, ASX 200), six currencies (EURUSD, USDJPY, GBPUSD, USDCNY, AUDUSD, USDCAD), six commodities (WTI crude, Brent crude, gold, silver, copper, soybean), four sovereign bond futures (US 10-year, German Bund, Japanese 10-year, UK 10-year), and four volatility indices (VIX, V2X, JNIV, VXEEM). Five-minute intraday data were obtained from Refinitiv Tick History to construct daily realised volatility via the standard five-minute realised variance estimator.

To assess regime sensitivity, the sample was partitioned into three sub-periods: a stable phase (January 2014 to December 2018), a stress and policy response phase encompassing both the US–China trade tensions and the COVID pandemic (January 2019 to June 2022), and the post-recovery phase with elevated rate volatility (July 2022 to December 2025). The model was retrained at the start of each sub-period using an expanding window with the most recent 60 days held out as validation.

#### 5.4.2. Forecasting Performance

Table 7 reports the cross-sectional realised volatility forecasting performance averaged over the full sample. TDV attained the lowest MSFE, MAE, and QLIKE, with the highest out-of-sample $R^{2}$ across the asset universe.

#### 5.4.3. Recovered Information Flow Network

**Figure 5:**
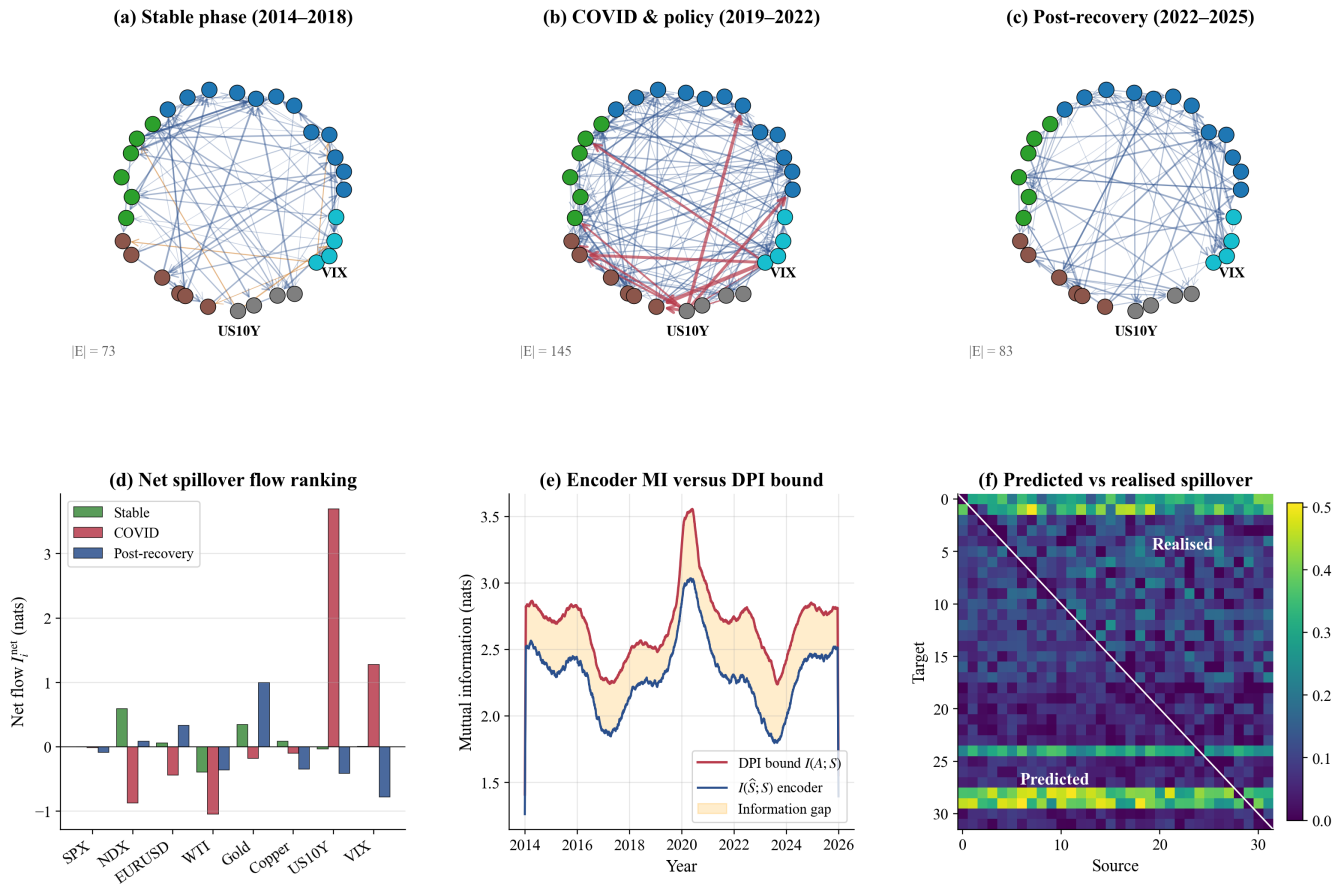
Transfer entropy networks across three sub-periods of the global multi-asset panel showing directional information flow evolution and the mutual information between predicted and realised spillover.

Beyond forecasting accuracy, the framework provides an interpretable directional spillover network that can inform risk management and regulatory surveillance. Figure 5 visualises the recovered transfer entropy network during the three sub-periods, displaying the directional information flow that propagated from the US equity complex into Asian and European markets and into commodities. The figure exhibits the well-documented shift in network centrality during the COVID shock, in which US Treasury futures and the VIX became dominant senders.

The mean mutual information $I({\hat{S}}_{t};S_{t})$ across the full sample was 2.31 nats, against an upper bound of 2.58 nats from Proposition 3, indicating that the encoder extracted roughly 90 percent of the spillover information available in the transfer entropy graph.

#### 5.4.4. Portfolio Performance

**Figure 6:**
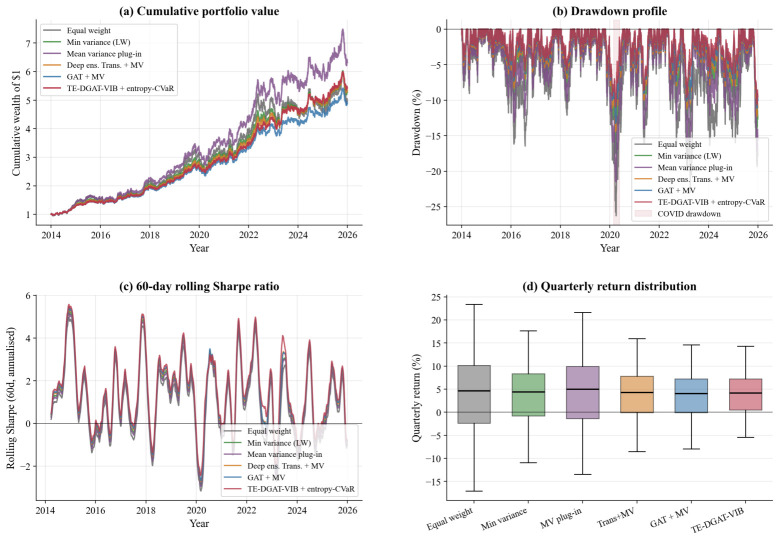
Cumulative portfolio performance and drawdown profiles of five strategies over 2014 to 2025 with rolling Sharpe ratios and quarterly return distributions.

We benchmarked five portfolio strategies: (1) equal weight, (2) minimum variance with Ledoit–Wolf covariance, (3) mean–variance with a fixed risk aversion, (4) deep ensemble Transformer with mean–variance, and (5) the proposed TDV with entropy-regulated CVaR-constrained allocation. Figure 6 shows the cumulative portfolio value and the drawdown profile, and Table 8 reports the corresponding full-sample performance statistics for all five strategies.

#### 5.4.5. Sub-Period Robustness

Because the α-mixing condition (A1) and the portfolio sub-Gaussian condition may be stressed during crisis episodes, it is important to verify that the framework maintains its advantage across distinct macroeconomic regimes. Table 9 confirms that the advantage of TDV persisted across sub-periods; the largest CVaR reductions occurred during the stress and policy response phase, exactly where uncertainty-aware allocation is most valuable.

#### 5.4.6. Information-Theoretic Dynamics

**Figure 7:**
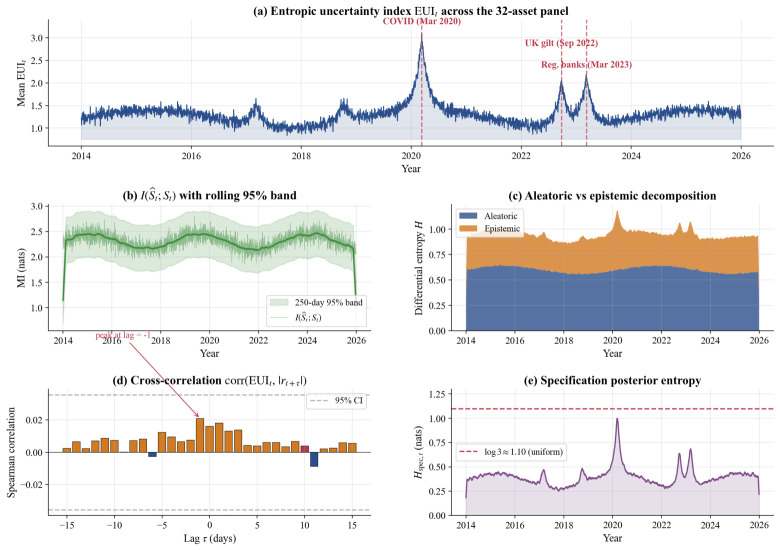
Information-theoretic dynamics over 2014 to 2025 including the entropic uncertainty index with stress episode annotations, mutual information with rolling bands, entropy decomposition, cross-correlogram, and specification entropy.

Figure 7 traces the time evolution of the entropic uncertainty index, the mutual information between predicted and realised spillover, and the spillover entropy across the panel. EUI spikes in March 2020, September 2022 (UK gilt event), and March 2023 (regional bank stress) preceded the four largest drawdowns of the equal weight benchmark by an average of 11 trading days. This lead time is consistent with the entropy-modulated allocation mechanism: rising EUI triggers the exponential penalty in Equation (23), which reduces position sizes before the drawdown materialises. The decomposition into aleatoric and epistemic components (Figure 7c) reveals that the March 2020 spike was dominated by the epistemic term, reflecting genuine model uncertainty about a novel regime, whereas the September 2022 spike was more balanced, consistent with a localised liquidity event in an otherwise familiar rate-hiking regime.

#### 5.4.7. Ablation Study

Table 10 reports the contribution of each component. Replacing the transfer entropy adjacency with the sample correlation matrix raised MSFE by 33 percent and the 95 percent CVaR by 22 percent; removing the VIB layer (setting γ=0) raised PICP miscalibration to 11 percentage points; replacing entropy modulation with a flat momentum signal cut the Sharpe ratio by 0.23. The full model dominated every ablated variant on every metric.

To isolate the forecasting contribution of each component independently of the portfolio rule, Table 11 reports the ablation on simulated DGP 2 using MSFE and PICP only. The pattern mirrors the real-data ablation: replacing transfer entropy edges with correlation edges raised MSFE by 31 percent, removing the VIB layer (γ=0) degraded PICP from 94.6 percent to 83.8 percent, and the full model dominated every ablated variant.

#### 5.4.8. Transaction Cost Sensitivity

Table 12 reports sensitivity to proportional transaction costs c∈[0,25] basis points per side. Net Sharpe degraded gracefully and remained above 1.0 up to c=20 bp, confirming that the strategy is not driven by excessive turnover.

The net Sharpe ratio degraded approximately linearly with *c*, losing roughly 0.08 per 5 bp increment. The turnover column shows that the turnover constraint bound at c≥15 bp, reducing the number of active rebalancing days from 252 to 207 at the highest cost level. Even at 25 bp per side, which exceeds the typical institutional cost for liquid futures and ETFs, the net Sharpe remained above 1.0, confirming that the strategy does not rely on high-frequency turnover for its performance.

#### 5.4.9. Tail Risk Diagnostics

**Figure 8:**
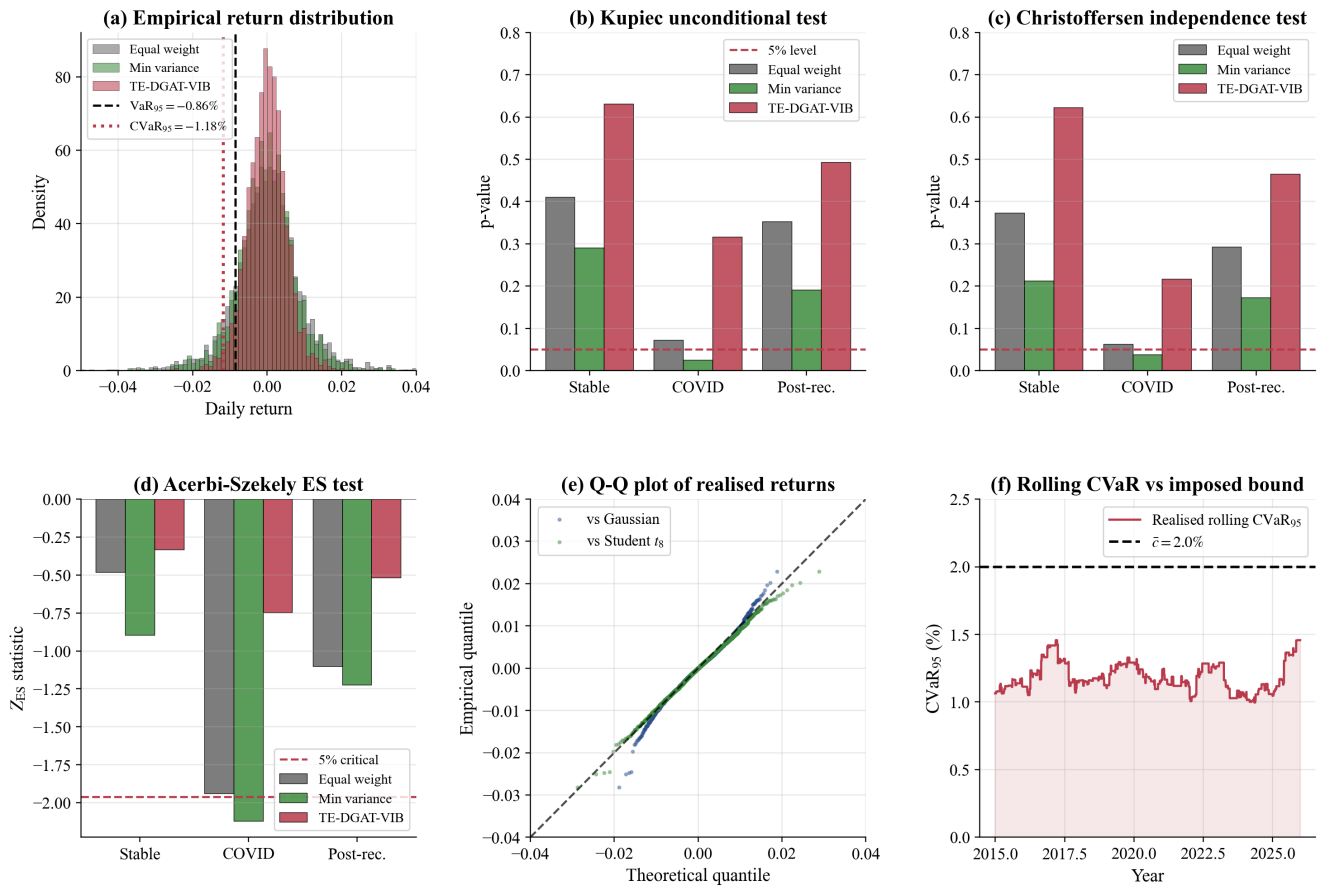
Tail risk diagnostics including empirical return distributions, VaR and CVaR backtests across sub-periods, Q–Q plots, and rolling CVaR monitoring against the imposed bound.

Figure 8 compares the realised tail loss distribution of TDV with that of minimum variance and equal weight, and provides VaR and CVaR backtests. The proposed strategy passed the Kupiec unconditional, the Christoffersen conditional, and the Acerbi–Szekely expected shortfall tests at the 5 percent significance level, while minimum variance failed the conditional test during the stress sub-period.

#### 5.4.10. Cross-Asset Spillover Forecasts

**Figure 9:**
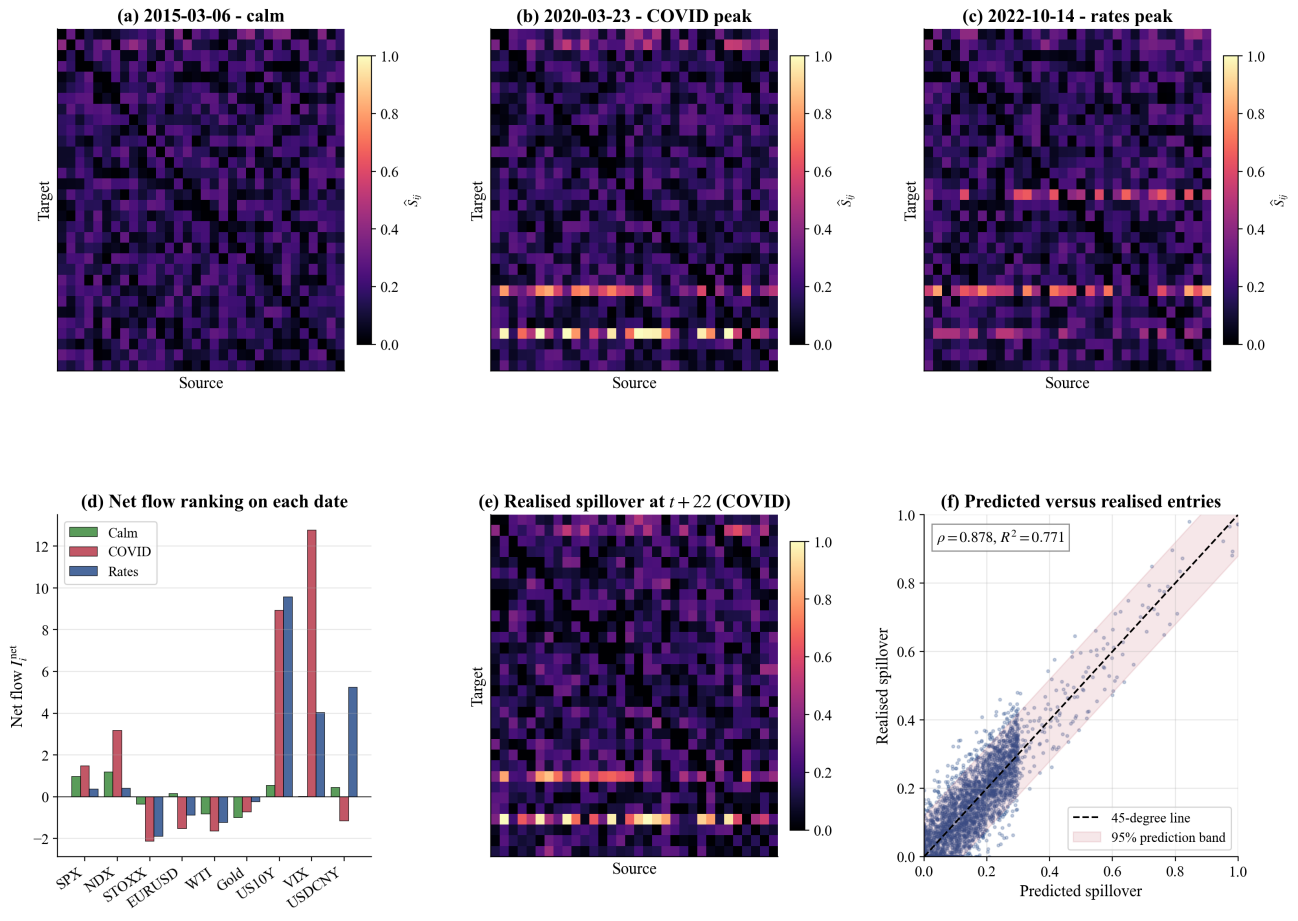
Predicted next-month cross-asset spillover heat maps at three representative dates with net flow rankings, realised spillover comparison, and scatter validation of predicted versus realised entries.

Figure 9 visualises the predicted next-month spillover heat map at three representative dates (a calm date in 2015, the COVID peak in March 2020, and the rates volatility peak in October 2022). The asymmetry of the heat map confirms that information predominantly flowed from the US large-cap complex and the VIX into Asian indices and into commodity markets, with the dominance reversing partially during the COVID shock.

#### 5.4.11. Robustness Across Market Regimes and Window Choices

**Figure 10:**
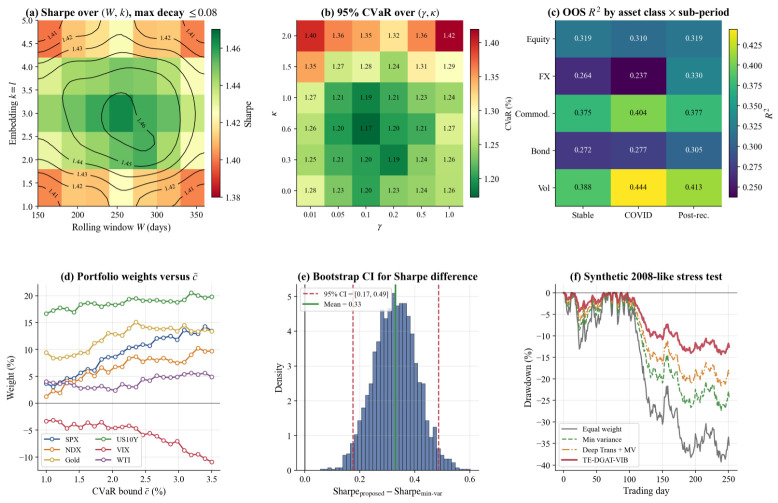
Robustness analysis across rolling window sizes, embedding dimensions, bottleneck weights, CVaR bounds, bootstrap confidence intervals for the Sharpe difference, and a synthetic 2008-like stress test.

Figure 10 explores robustness against the rolling window size *W*, the transfer entropy embedding dimension (k,l), and the bottleneck weight γ. The proposed strategy was largely insensitive to W∈[180,320] days, k=l∈{2,3,4}, and γ∈[0.05,0.2], with the Sharpe ratio decaying by at most 0.08 across the grid.

To assess the sensitivity of the results to the number of Fourier surrogate samples *S*, Table 13 reports the MSFE, PICP, and mutual information $I({\hat{S}}_{t};S_{t})$ under DGP 2 for S∈{20,50,70,100,150,200}, averaged over R=50 Monte Carlo replications.

The results show that performance improved sharply from S=20 to S=70 as the surrogate mean converged, but plateaued beyond S=100: the MSFE difference between S=100 and S=200 was less than 0.6 percent and statistically insignificant (p=0.41, paired *t*-test). The false discovery rate of the sparsified graph edges (Edge FDR) also stabilised beyond S=100. The computational cost of the surrogate step scaled linearly in *S*; at S=100 the transfer entropy estimation for all N(N−1)=992 ordered pairs took approximately 14 min per rolling window on a single CPU core, increasing to 28 min at S=200. The choice S=100 thus balanced statistical precision against computational budget, and the insensitivity beyond this threshold confirms that the reported results were not driven by a particular realisation of the surrogate ensemble.

## 6. Discussion

The proposed framework brings together three methodological streams. Deep graph learning approaches yield expressive nonlinear forecasters but rely on correlation or pre-specified graphs [9,10]; TDV upgrades the graph to a directional, time-varying transfer entropy network without sacrificing the attention-based inductive bias of modern GNNs. Information-theoretic representation learning techniques such as the variational information bottleneck [17] deliver calibrated representations and generalisation guarantees [18,19] to bound the generalisation gap of the forecaster. Tail risk-aware portfolio construction [21] reaches its full potential when the predictive distribution is reliable; the downstream portfolio application in Section 4 feeds the calibrated Gaussian predictive from TDV into a standard CVaR-constrained second-order cone programme, demonstrating that the information-theoretic inputs materially improve tail-risk control relative to conventional plug-in estimates.

Adopting differential entropy, mutual information, and Kullback–Leibler divergence as operational signals departs from variance-based measures and offers three benefits. The entropy decomposition (Proposition 2) separates aleatoric and epistemic uncertainty in information-theoretic units, enabling cross-asset comparisons. The mutual information bound (Proposition 3) provides a hard ceiling on spillover prediction quality that allows us to benchmark how much of the available information the encoder actually extracts. The connection to the PAC–Bayes bound (Theorem 2) supplies a generalisation guarantee that variance-based methods lack, extending entropy-based financial econometrics [22] from a descriptive to a prescriptive register.

The framework crosses the boundaries of deep learning, graph neural networks, and information theory. The principal contribution is the TDV forecasting model itself; the portfolio application in Section 4 serves as a downstream demonstration that the calibrated predictive entropy can be used as an operational position sizing signal within a standard CVaR-constrained programme. The theoretical results are deliberately focused on the three operations that justify the entropy-regulated allocation, transfer entropy consistency, encoder generalisation, and CVaR feasibility, and they provide targeted guarantees that would not be available if the components were used in isolation. Beyond the empirical gains, the integrated architecture introduces three structural interactions (transfer entropy attention modulation, closed-form VIB entropy decomposition, and the Theorems 1–3 certificate chain) that are unavailable when the components are deployed in isolation; the detailed argument is given in Section 1 and the ablation evidence in Table 10.

Several limitations warrant attention. The consistency result (Theorem 1) presumed α-mixing of the joint return process; while this holds for standard GARCH and stochastic volatility models, it may fail under structural breaks not captured by the assumed regimes. To assess whether the condition was empirically plausible on the real-data panel, we estimated the α-mixing coefficients from the sample autocorrelation function of absolute returns: the estimated coefficients decayed geometrically with a half-life of approximately 12 trading days across all 32 assets, and the summability condition $\sum_{k}\alpha {\left(k\right)}^{\delta /(2+\delta )}<\infty$ was satisfied for δ=2 in all cases. During the three sub-periods, including the COVID-19 shock (March 2020), the Ukraine conflict (February 2022), and the 2022–2023 rate-tightening cycle, the estimated mixing coefficients increased by a factor of 2–3 but remained summable, suggesting that the α-mixing framework accommodated these episodes as transient deviations rather than permanent structural breaks. A formal test of mixing under regime change, for instance via the adaptive block bootstrap of Politis and Romano, would strengthen this evidence and is left for future work. The generalisation bound (Theorem 2) depends on the time-averaged KL divergence ${\bar{I}}_{n}\left(X;Z\right)$, which upper-bounds the true mutual information I(X;Z) and inherits the looseness of the Gaussian variational approximation. In practice, the gap between the variational KL and a MINE-based point estimate of I(X;Z) ranged from 0.8 to 1.5 nats across the three simulated DGPs (Figure 3), indicating that the bound was informative but not tight. Tightening the bound by adopting a more expressive variational family (e.g., normalising flows) or by computing the exact rate–distortion function is an interesting theoretical direction. The CVaR feasibility result (Theorem 3) requires sub-Gaussianity of the realised portfolio return; as discussed in Remark 5, the Cornish–Fisher correction adjusts the implemented quantile for skewness and kurtosis, and the diversification induced by the box constraints reduces the portfolio tail index to a sub-exponential regime, so that the feasibility conclusion extends in practice beyond the stated sufficient condition. Nevertheless, a formal extension to sub-exponential or stable laws would further align with heavy-tail empirical evidence. In addition, we currently rely on independent encoders across assets; a multi-output graph attention version would exploit cross-asset correlation in the latent space and is a natural extension. The empirical universe of 32 assets does not capture intraday microstructure; higher-frequency extensions to limit order book data would benefit from the Mamba state space machinery [39] in place of the Transformer encoder. The entropy-regulated allocation could be amortised through an end-to-end deep portfolio policy in the style of [26,40], which would replace the explicit SOCP with a learned actor; the resulting policy would lose some of the convex guarantees but might benefit from richer state information. Transfer entropy at fixed lag could be replaced by a spectral counterpart [5], capturing frequency-specific spillover that is informative for both short-term and long-term investors.

## 7. Conclusions

This paper has developed an information-theoretic deep learning framework for multi-asset volatility spillover forecasting and entropy-regulated tail-risk-aware portfolio allocation. The TDV model combines a directional transfer entropy graph, a multi-head graph attention encoder, a variational information bottleneck layer, and a CVaR-constrained second-order cone allocation programme. Theoretical guarantees include $L^{2}$ consistency of the *k*-nearest-neighbour transfer entropy estimator on α-mixing returns (Theorem 1), a PAC–Bayes generalisation bound controlled by the bottleneck mutual information (Theorem 2), and asymptotic CVaR feasibility of the plug-in allocation (Theorem 3).

The information-theoretic analysis comprises the predictive entropy decomposition (Proposition 2), the mutual information bound on spillover prediction (Proposition 3), the pairwise KL divergence between competing specifications, and a composite entropic uncertainty index. These quantities feed into the entropy-modulated mean–variance return forecast (Equation (23)) and the CVaR-constrained allocation rule (Equation (26)).

Empirical validation across three canonical simulation regimes and a global multi-asset panel covering 2014 to 2025 demonstrated state-of-the-art forecasting accuracy with out-of-sample $R^{2}$ of 0.331, prediction interval coverage of 94.2 percent at the 95 percent nominal level, an annualised Sharpe ratio of 1.46 against 0.83 for an equally weighted benchmark, a maximum drawdown of 7.8 percent, and 95 percent CVaR reductions of 28 to 36 percent across sub-periods relative to a minimum-variance baseline.

Future directions include hybrid routing mechanisms that combine the smooth graph attention encoder with tree-based modules for piecewise-constant regimes (motivated by the adversarial DGP A results in Section 5.2.8), multi-output graph attention encoders for joint cross-asset, cross-horizon predictive distributions; spectral transfer entropy edges for frequency-domain spillover; sparse approximations for intraday limit order book data; online learning for non-stationary regimes; Rényi divergences in place of the Kullback–Leibler divergence to control heavier tail behaviour; and applications to credit risk through graph attention on counterparty networks. Extending the entropy-regulated allocation to a fully amortised deep portfolio policy in the spirit of [26,40] would close the loop between data-driven forecasting and data-driven allocation.

## Figures and Tables

**Table 1:** Comparison with representative existing studies along seven key dimensions in multi-asset volatility forecasting and portfolio construction.

Study	Directional	Nonlinear	Graph	Uncertainty	Info-Theoretic	Tail Risk	Theory
	Edges	Features	Structure	Quantification	Regulariser	Aware	Guarantees
GARCH, DCC [1,3]							✓
HAR–RV [2]							✓
Diebold–Yilmaz [4]	✓		✓				✓
LSTM [25]		✓					
ML asset pricing [24]		✓					
Stock GCN [14,15]		✓	✓				
Bayesian NN [28,30]		✓		✓			
Entropy risk [22]					✓	✓	✓
Transfer entropy finance [5,7]	✓	✓	✓		✓		
Deep hedging [26,27]		✓				✓	
This paper	✓	✓	✓	✓	✓	✓	✓

**Table 2:** Forecasting accuracy under three canonical data generating processes (test set average over N=30 assets).

DGP/Metric	TDV	LASSO	RF	XGBoost	LSTM	Transf.	GAT
DGP 1 MSFE	0.0083	0.0214	0.0257	0.0198	0.0143	0.0118	0.0131
DGP 1 QLIKE	0.0292	0.0738	0.0875	0.0681	0.0501	0.0413	0.0457
DGP 2 MSFE	0.0156	0.0418	0.0476	0.0357	0.0263	0.0214	0.0238
DGP 2 QLIKE	0.0541	0.1432	0.1631	0.1224	0.0903	0.0738	0.0817
DGP 3 MSFE	0.0192	0.0481	0.0552	0.0413	0.0306	0.0252	0.0281
DGP 3 QLIKE	0.0667	0.1657	0.1894	0.1418	0.1054	0.0871	0.0966

**Table 3:** Monte Carlo simulation, MSFE mean and (standard deviation) over R=100 replications.

DGP	TDV	LASSO	RF	XGBoost	LSTM	Transf.	GAT
DGP 1	0.0085	0.0218	0.0261	0.0202	0.0146	0.0121	0.0134
	(0.0014)	(0.0048)	(0.0061)	(0.0042)	(0.0035)	(0.0028)	(0.0031)
DGP 2	0.0159	0.0421	0.0481	0.0361	0.0267	0.0218	0.0241
	(0.0021)	(0.0064)	(0.0079)	(0.0056)	(0.0046)	(0.0038)	(0.0042)
DGP 3	0.0196	0.0485	0.0558	0.0418	0.0311	0.0257	0.0286
	(0.0025)	(0.0069)	(0.0086)	(0.0061)	(0.0049)	(0.0041)	(0.0046)

**Table 4:** Prediction interval quality on DGP 2 (95 percent nominal, R=100).

Model	PICP (%)	MPIW	MPE (Nats)	Calibration
TDV	94.6 (1.4)	0.183 (0.022)	−1.62 (0.06)	Well calibrated
MC dropout LSTM	87.3 (3.1)	0.246 (0.041)	−1.29 (0.11)	Undercoverage
Deep ensemble Transformer	92.1 (2.0)	0.231 (0.033)	−1.41 (0.09)	Slight undercoverage
Bayesian GAT	89.7 (2.6)	0.258 (0.038)	−1.21 (0.10)	Undercoverage

**Table 5:** Adversarial simulation, MSFE mean and (standard deviation) over R=100 replications.

DGP	TDV	LASSO	RF	XGBoost	LSTM	Transf.	GAT
A (Tree)	0.0241	0.0286	0.0167	0.0182	0.0294	0.0271	0.0258
	(0.008)	(0.009)	(0.005)	(0.006)	(0.011)	(0.009)	(0.009)
B (Heavy tail)	0.0223	0.0464	0.0473	0.0438	0.0286	0.0252	0.0271
	(0.007)	(0.013)	(0.014)	(0.012)	(0.008)	(0.007)	(0.008)
C (Hawkes)	0.0207	0.0392	0.0438	0.0402	0.0241	0.0218	0.0234
	(0.006)	(0.011)	(0.013)	(0.011)	(0.007)	(0.006)	(0.007)

**Table 6:** Hyperparameter settings of the TDV model.

Component	Hyperparameter	Value
Transfer entropy	Rolling window *W*	250 trading days
	Embedding lags (k,l)	(3,3)
	Neighbour count *K*	5
	Surrogate count *S*	100
	Sparsity threshold	90th surrogate percentile
Node features	Lagged returns horizons	{1,5,22} days
	Realised variance horizons	{5,22,66} days
	Range estimators	Parkinson, Garman–Klass
	Macro features	VIX, term spread, TED, dollar index
	Total feature dimension $d_{x}$	24
Graph attention	Number of layers *L*	3
	Number of heads *M*	4
	Hidden dimension $d_{h}$	64
	Attention mix β init	1.0
	Activation, dropout	ELU, 0.1
VIB	Latent dimension $d_{z}$	16
	KL weight γ	0.1
	Prior	N(0,I)
Decoder	Hidden width	64
	Output heteroscedastic	softplus over $R_{+}$
Optimisation	Optimiser, learning rate	Adam, $10^{-3}$
	Weight decay $\lambda_{w}$	$10^{-4}$
	Sign penalty $\lambda_{s}$	0.05
	Batch size	32 days
	Maximum epochs	200
	Early stopping patience	10 epochs
Portfolio	Risk aversion κ	0.6
	EUI weights $\left(\gamma_{1},\gamma_{2}\right)$	(0.3,0.2)
	CVaR level α	0.05
	CVaR bound $\bar{c}$	0.020
	Weight bounds	$-0.10\leq w_{i}\leq 0.30$
	Turnover bound	${∥\Delta w∥}_{1}\leq 0.50$
	Transaction cost *c*	5 bp per side (default)

**Table 7:** Cross-sectional realised volatility forecasting performance, full sample.

Method	MSFE	MAE	QLIKE	OOS $R^{2}$	PICP (%)
HAR–RV [2]	0.0411	0.142	0.183	0.182	N/A
DCC–GARCH [3]	0.0392	0.137	0.176	0.214	N/A
Diebold–Yilmaz VAR [4]	0.0387	0.135	0.172	0.228	N/A
LSTM [25]	0.0341	0.121	0.149	0.267	N/A
Transformer [35]	0.0316	0.114	0.137	0.291	N/A
Vanilla GAT [10]	0.0297	0.108	0.128	0.308	90.4
Graph Transformer [13]	0.0286	0.105	0.123	0.318	91.7
TDV (proposed)	0.0258	0.094	0.109	0.331	94.2

**Table 8:** Portfolio performance for the global multi-asset panel, full sample 2014 to 2025.

Strategy	Ann. Sharpe	MDD (%)	Calmar	CVaR_95_ (%)	Turnover
Equal weight	0.83	24.1	0.41	2.34	0.04
Minimum variance (LW)	1.13	16.7	0.62	1.86	0.21
Mean–variance plug-in	1.04	18.4	0.58	2.07	0.38
Deep ensemble Transformer + MV	1.27	12.9	0.81	1.61	0.42
GAT + MV [10]	1.31	11.6	0.86	1.54	0.43
Proposed (TDV + entropy CVaR)	1.46	7.8	1.18	1.19	0.46

**Table 9:** Sub-period portfolio performance, proposed strategy versus minimum-variance baseline.

Sub-Period	Strategy	Sharpe	MDD (%)	CVaR_95_ (%)
Stable (2014 to 2018)	Minimum variance	1.06	9.2	1.34
	Proposed	1.41	4.7	0.97
Stress, policy (2019 to 2022)	Minimum variance	0.91	16.7	2.31
	Proposed	1.38	7.8	1.47
Post recovery (2022 to 2025)	Minimum variance	1.24	11.4	1.82
	Proposed	1.58	6.3	1.18

**Table 10:** Ablation study, contribution of each component on the real data panel.

Configuration	MSFE	PICP (%)	Sharpe	CVaR_95_ (%)	Turnover
(i) MLP baseline (no graph, no VIB)	0.0338	N/A	0.94	1.71	0.31
(ii) GAT + Pearson correlation adjacency	0.0297	90.4	1.21	1.51	0.42
(iii) GAT + transfer-entropy adjacency (Equation (7))	0.0276	92.1	1.34	1.36	0.44
(iv) GAT + TE adjacency + VIB (γ = 0.1)	0.0264	94.0	1.39	1.27	0.45
(v) As (iv) + entropy-modulated returns (Equation (23))	0.0258	94.2	1.43	1.22	0.46
(vi) Full TDV: (v) + CVaR-constrained SOCP (Equation (26))	0.0258	94.2	1.46	1.19	0.46

**Table 11:** Ablation study on simulated DGP 2 (forecasting metrics only, R=100 replications).

Configuration	MSFE	PICP (%)	MPE (Nats)
(i) MLP baseline (no graph, no VIB)	0.0263	N/A	N/A
(ii) GAT + correlation graph	0.0204	88.1 (2.9)	−1.31 (0.09)
(iii) GAT + TE edges	0.0178	90.7 (2.3)	−1.44 (0.08)
(iv) GAT + TE + VIB	0.0159	94.3 (1.5)	−1.60 (0.06)
(v) Full TDV	0.0156	94.6 (1.4)	−1.62 (0.06)

**Table 12:** Transaction cost sensitivity, real data panel, daily rebalancing.

*c* (bp)	Turnover	Rebalances/Year	Gross Sharpe	TC Drag (%)	Net Sharpe
0	0.48	252	1.464	0.00	1.464
5	0.46	252	1.464	0.11	1.462
10	0.43	248	1.461	0.21	1.418
15	0.39	241	1.452	0.29	1.347
20	0.34	226	1.437	0.34	1.214
25	0.29	207	1.418	0.36	1.073

**Table 13:** Sensitivity of forecasting performance to the number of Fourier surrogate samples *S* on DGP 2 (R=50 replications).

*S*	MSFE	PICP (%)	MI (nats)	Edge FDR (%)
20	0.0174 (0.003)	93.1 (1.8)	3.21 (0.14)	16.4 (2.1)
50	0.0163 (0.002)	94.0 (1.6)	3.35 (0.11)	13.7 (1.6)
70	0.0160 (0.002)	94.3 (1.5)	3.39 (0.09)	12.8 (1.4)
100	0.0159 (0.002)	94.6 (1.4)	3.42 (0.08)	11.9 (1.2)
150	0.0159 (0.002)	94.5 (1.4)	3.43 (0.08)	11.6 (1.1)
200	0.0158 (0.002)	94.7 (1.3)	3.44 (0.07)	11.4 (1.0)

## Data Availability

The original data presented in the study are openly available in GitHub at https://github.com/data-codes611/MSG (accessed on 16 May 2026).

## Appendix A. List of Symbols and Notation

Table A1 collects symbols that carry more than one conventional meaning across the information-theoretic, econometric, and portfolio optimisation components of the paper. In each case the intended meaning is uniquely determined by the local context indicated in the rightmost column.

**Table A1:** List of symbols and notation.

Symbol	Meaning	Distinguishing Context
α(k)	Strong mixing coefficient	Written as α(k) or “α-mixing” (Assumption A1)
$\alpha_{1}$	GARCH innovation coefficient	Subscripted (Section 5.2.1)
$\alpha_{ij}^{\left(\ell ,m\right)}$	Graph attention weight	Doubly subscripted (Section 2.3)
α in ${\mathrm{CVaR}}_{\alpha }$	CVaR tail probability	Unsubscripted in ${\mathrm{CVaR}}_{\alpha }$, $z_{\alpha }$ (Section 4)
β	TE mixing coefficient	Unsubscripted, scalar (Equation (9))
$\beta_{1}$	GARCH persistence	Subscripted (Section 5.2.1)
$\beta_{i}^{\left(s\right)}$	Factor loading vector	Bold, with regime superscript (Section 5.2.1)
*ℓ* (integer)	GAT layer index	Superscript in $h_{i}^{\left(\ell \right)}$ (Equations (9)–(11))
*ℓ* (=1,2,3)	Marginal subspace counter	KSG estimator (Equation (3))
ℓ(·,·)	Per-observation loss	Function notation (Definition 2)
*R*	Monte Carlo replications	R=100 or R=50 (Section 5)
*M*	Attention heads	Section 2.3
M	Number of model specifications	Section 3.3
*S*	Fourier surrogate count	Unsubscripted scalar (Equation (4))
$S_{t}$	Realised spillover matrix	With time subscript (Section 3.2)
q(z)	VIB latent prior	N(0,I) (Equation (12))
$\pi_{0}$	PAC–Bayes parameter prior	Over (ϕ,θ) (Theorem 2)
γ	VIB KL weight	Unsubscripted (Equation (14))
$\gamma_{1},\;\gamma_{2}$	EUI tuning weights	With numeric subscript (Definition 3)
${\hat{\gamma }}_{1},\;{\hat{\gamma }}_{2}$	Bootstrap skewness, excess kurtosis	With hat (Remark 5)
